# Financing Healthcare in Central and Eastern European Countries: How Far Are We from Universal Health Coverage?

**DOI:** 10.3390/ijerph18041382

**Published:** 2021-02-03

**Authors:** Marzena Tambor, Jacek Klich, Alicja Domagała

**Affiliations:** 1 Department of Health Economics and Social Security, Institute of Public Health, Faculty of Health Sciences, Jagiellonian University Medical College, 31-008 Krakow, Poland; 2 Department of Public Management, Cracow University of Economics, 31-510 Krakow, Poland; uuklich@cyf-kr.edu.pl; 3 Department of Health Policy and Management, Institute of Public Health, Faculty of Health Sciences, Jagiellonian University Medical College, 31-008 Krakow, Poland; alicja.domagala@uj.edu.pl

**Keywords:** healthcare financing, universal health coverage, Central and Eastern European countries

## Abstract

After the fall of communism, the healthcare systems of Central and Eastern European countries underwent enormous transformation, resulting in departure from publicly financed healthcare. This had significant adverse effects on equity in healthcare, which are still evident. In this paper, we analyzed the role of government and households in financing healthcare in eight countries (EU-8): Czechia, Estonia, Hungary, Latvia, Lithuania, Poland, Slovakia, and Slovenia. A desk research method was applied to collect quantitative data on healthcare expenditures and qualitative data on gaps in universal health coverage. A linear regression analysis was used to analyze a trend in health expenditure over the years 2000–2018. Our results indicate that a high reliance on out-of-pocket payments persists in many EU-8 countries, and only a few countries have shown a significant downward trend over time. The gaps in universal coverage in the EU-8 countries are due to explicit rationing (a limited benefit package, patient cost sharing) and implicit mechanisms (wait times). There is need to increase the role of public financing in CEE countries through budget prioritization, reducing patient co-payments for medical products and medicines, and extending the benefit package for these goods, as well as improving the quality of care.

## 1. Introduction

The economic and political transformation in postsocialist countries initiated after 1989 has gotten an impressive body of literature [1,2,3,4,5,6]. Transformation processes in Central and Eastern European (CEE) countries are among the most significant events of the end of the twentieth century [7]. An important component of transformation in postsocialist countries was the change of the healthcare system. The reforms following the collapse of communism, provoked by a sharp economic decline, resulted in departure from the centralized and nationalized healthcare systems of the Semashko model [8,9,10]. The scope of transformation was wide, starting from a shift in ownership (transforming public entities into private ones and/or establishing private healthcare entities), through changes in organization (disintegration of care and strengthening primary healthcare), and ending up with changes in healthcare financing (introduction of social health insurance in most CEE countries) [9,10,11].

It was expected that the shift towards an insurance-based system with market-oriented features in CEE countries would ensure sufficient and more stable funds for healthcare and improve efficiency (also through increasing patient responsibility for financing healthcare) [8,9,10]. The reforms were, however, seldom based on evidence, and they suffered from institutional shortcomings, e.g., insufficient contribution rates or poor effectiveness in collection of contributions [8,9,12]. To add to this, their implementation encountered difficult economic condition. Thus, the outcomes of the reforms were far from expected. Patient out-of-pocket payments grew significantly, increasing inequality in healthcare systems [13]. Households were made responsible for financing healthcare though formal cost sharing [14,15,16]. On top of this, informal patient payments, already present during the communist era, became even more widespread during the transition period [17,18].

Currently, 30 years after the collapse of communism, CEE countries are still struggling to ensure sufficient public resources for health and catch up with Western European countries in ensuring universal health coverage, i.e., equal access to necessary healthcare without financial hardship for patients [19]. The economic crisis of 2008 stood in the way, strongly affecting many CEE countries and perpetuating differences between east and west Europe [20]. The current crisis caused by the COVID-19 pandemic might similarly have detrimental effects on healthcare systems. The future also holds challenges for the countries of this region. Rapid population ageing due to particularly low fertility rates, in addition to migration, will very likely deepen the fiscal imbalance in health budgets of the CEE countries [21].

In this paper, we analyzed the role of the government and households in financing healthcare in eight CEE countries: Czechia, Estonia, Hungary, Latvia, Lithuania, Poland, Slovakia, and Slovenia. These countries were the first in the CEE region to join the European Union (EU) in 2004 (referred to as the EU-8). We have analyzed quantitative data on healthcare expenditure since 2000, as well as qualitative data on the gaps in public healthcare coverage in terms of population and services excluded from coverage, as well as patient obligation to share the cost of publicly financed healthcare. This allowed us to see what directions CEE countries are moving in, regarding government responsibility for financing healthcare, and indicate the obstacles to achieving universal health coverage—one of the health-related United Nations sustainable development goals.

The details on the methods applied in this study are presented in the next section. This is followed by the results presented separately for quantitative and qualitative data analysis. The last two sections include discussion of the results and conclusions drawn from our research.

## 2. Materials and Methods

This study aimed to analyze the role of government and households in financing healthcare in the EU-8 countries. All of the included countries share similar experiences with the Siemasko healthcare system, and they all began a series of healthcare reforms in the 1990s, although it should be noted that there have been differences across the countries, e.g., in the extent of the undertaken reforms, their speed, and the economic and social environment of the health systems. At present, all these countries, with the exemption of Latvia, rely on social health insurance to collect resources for healthcare, though only Czechia and Slovakia have a competitive insurance model [22,23,24,25,26,27,28,29]. The countries differ significantly in demographic, social, and economic characteristics, with Latvia having the smallest population, the highest old-age dependency ratio, and the lowest GDP per capita. Slovenia and Czechia are the most developed countries with the highest GDP per capita and the highest Human Development Index score, as well as the longest life expectancy. The World Bank’s governance effectiveness index is rather low in all of the analyzed countries, particularly in Hungary and Poland. Across the countries, there is close to universal support for the state being responsible for ensuring access to healthcare. For details, see Table 1.

To meet the aim of the study, a desk research method has been applied. A narrative literature review of databases and publications by the Organization for Economic Co-operation and Development (OECD), the World Health Organization (WHO), and the EU, was conducted between May 2020 and January 2021 to identify and collect quantitative and qualitative data on healthcare expenditure and healthcare coverage by government schemes in the EU-8 countries.

The quantitative component of our study relied on data on healthcare expenditure from the National Health Accounts available in OECD and WHO databases [33,34]. We selected five health expenditure indicators, which enabled a comprehensive analysis of healthcare financing with the focus on the respective roles of government and households, namely:

- Current health expenditure as % of GDP—this presents total spending on healthcare goods and services (excluding investment spending) by all types of financing arrangements (compulsory schemes, household out-of-pocket payments, voluntary health insurance, non-governmental organizations etc.) in relation to country GDP. Source of data: OECD Health Statistics (accessed on 20 July 2020) [33].
- Domestic general government health expenditure as % of general government expenditure—this compares the scale of current public health expenditure (by all financing agents holding public domestic funds) relative to the total scale of government expenditure. Thus, it indicates the government’s priority to spend on health out of its own domestic public resources. Source of data: World Health Organization Global Health Expenditure Database (accessed on 10 January 2021) [34].
- Government/compulsory scheme expenditure as % of current health expenditure—this presents the share of government (central and regional/local) and compulsory financing schemes (e.g., compulsory health insurance) in the total current expenditure. This mostly includes spending by government and social health insurance, though compulsory private health insurance is also included if present. Source of data: OECD Health Statistics (accessed on 20 July 2020) [33].
- Household out-of-pocket health expenditure as % of current health expenditure—out-of-pocket expenditures are payments borne directly by patients when using healthcare. They include direct payments for privately purchased healthcare (without involvement of third-party payers, e.g., an insurer) and patient cost sharing for goods and services covered by third-party payers. This also includes estimations of informal payments to healthcare providers when such data are available. Source of data: OECD Health Statistics (accessed on 20 July 2020) [33].
- Household out-of-pocket expenditure as % of health expenditure for given types of services—this gives more detailed information on household contributions to financing healthcare, and indicates the areas where public coverage is limited. Source of data: OECD Health Statistics (accessed on 20 July 2020) [33].

To present the changes in health expenditure over the years, in our analysis we have considered a time span of 19 years (from 2000 until 2018). In order to explore whether a trend is evident in health expenditure data over the years, we applied a linear least squares regression analysis (using MS Excel 2016, and R version 4.0.3), where time was used as an explanatory variable. The analysis was performed for each country as well for the whole group, including countries’ variables in the model.

In order to have a better picture of household involvement in financing healthcare, we also examined more in-depth information on healthcare coverage by government schemes and compulsory insurance schemes. We considered three dimensions of coverage:

- Population coverage—population entitlement to a benefit package, financed from government or compulsory insurance schemes;
- Service coverage—the range of goods and services included in a benefit package. We focused on healthcare benefits (primary care, outpatient and inpatient specialist care, dental care, medical products, medicines), excluding sickness benefits or maternity benefits (even if in some countries they are financed though the obligatory health insurance fund). When analyzing service coverage, we also looked at the quality of services in a benefit package. Quality might be considered the fourth dimension of healthcare coverage, as gaps in this aspect might also lead to out-of-pocket patient payments.
- Cost coverage—patient cost-sharing obligations for healthcare in a benefit package. This might include a) flat-rate payments (co-payments) per good or service; b) percentage co-payments (sometimes referred to as co-insurance) when a patient pays a share of the price; c) deductibles, which require users to pay up to a fixed amount first, before the state/insurer will cover any costs. Patients might be also asked to cover any cost over the amount of money reimbursed by the insurer/state if the price of service or good exceeds the reimbursement amount (balance billing/extra billing/reference pricing).

Data for this qualitative analysis have largely been obtained from the country and cross-country reports found on the websites of the OECD, WHO, and EU, which allow us to ensure a relatively high degree of comparability of data between countries. These key reports have been selected:

- The Health Systems in Transition (HiT) country profiles by the European Observatory on Health Systems and Policies;
- The State of Health in the EU country profiles by the OECD and the European Observatory on Health Systems and Policies;
- Universal Health Coverage: Financial Protection Country Reviews by the WHO Regional Office for Europe;
- The Health at a Glance report series by the OECD and the EU.

We cross-checked data from these various sources in order to assure their validity. They are mostly of a qualitative nature, though, if available, quantitative data on healthcare coverage were also collected. We mostly present the current situation in the countries, yet we also attempt to outline the changes in policies related to coverage over the years.

## 3. Results

In this section, we first analyzed the data on healthcare expenditure in the EU-8 countries. Then, we present the results of the qualitative data analysis of the dimensions of universal health coverage, i.e., population entitlement universality, coverage of services in the benefit package, and quality and access to these services, as well as patient cost-sharing obligations.

### 3.1. Healthcare Expenditure

Table 2 presents data on the four health expenditure indicators for each country, including their value at the beginning and at the end of the analyzed period, the minimum and maximum annual growth rate (percentage change over previous year) during this period, and the estimated simple linear regression model to examine the trend in health expenditure data over the years. The definitions of the indicators are included in the methods section, while the Supplementary Materials (Figures S1–S32 and Table S1) include a full dataset on health expenditure and a graphical presentation of linear regression results.

The presented data show great diversity in healthcare expenditure indicators across the countries. The annual growth rates also indicate that there were falls and rises in their levels during the analyzed period.

In 2018, current healthcare expenditure as percentage of GDP ranged from 6.2% in Latvia to 8.3% in Slovenia. Since 2000, the expenditure has increased in all countries but Hungary. Data suggest that Hungary has been marked with a small decrease of current health expenditure as share of GDP, though this was from a relatively high level, i.e., 6.8% in 2000. The results of linear trend regression analysis show a statistically significant linear trend for all countries but Latvia and Hungary, with the highest average annual increase in Estonia, where the rate of current health expenditure as percentage of GDP has increased on average by 0.11 annually. In some of the countries, the sharpest rise in the value of the indicator occurred during the economic crisis of 2008, which can be attributed to a drop in the GDP level.

Domestic general government health expenditure as percentage of general government expenditure in 2018 ranged from 9.6% in Latvia to 15.5% in Czechia. Data indicate that since 2000, all governments have increased the priority given to healthcare when making decisions regarding public expenditure, with the exception of Hungary. Yet, a statistically significant upward trend was only observed in Czechia, Estonia, Poland, and Slovakia. The highest increase in the share of public expenditure devoted to health between 2000 and 2018 was in Slovakia (by 3.8 p.p.), albeit from the low level of 9%.

The data on the contribution of public spending and household out-of-pocket payments in health expenditure showed great diversity across the countries. The differences observed in 2000 have persisted until recent years as countries have moved in different directions. Among the eight countries, Czechia and Slovakia are characterized by the highest share of public spending in current health expenditure (83% and 80%, respectively, in 2018). Public involvement in financing healthcare in Czechia and Slovakia was already high in 2000 (nearly 90%) and these two countries have been marked with the highest decrease in the public share (by 7 and 9 p.p. in Czechia and Slovakia, respectively) and an increase in the out-of-pocket share in financing healthcare by 4 and 8 p.p. respectively. In Czechia, the decreasing trend for public spending and the increasing trend for out-of-pocket payments were statistically significant, while in Slovakia no stable trends could be seen. At the opposite end is Latvia, with the lowest share of public financing and the highest share of out-of-pocket payments (60% and 39%, respectively, in 2018). The situation in Latvia has improved throughout the years, with a statistically significant increasing trend of the rate of the public share in healthcare expenditure, on average by 0.5 per year, and the highest overall growth among all the countries, i.e., by 9 p.p. between 2000 and 2018.

In all other countries (Lithuania, Hungary, Poland, Slovenia, Estonia) the public share in healthcare spending ranged from 67% to 74% (2018) and there were rather small changes between 2000 and 2018 (+/– 3 pp), though a significant decreasing trend in Hungary, Lithuania, and Estonia might raise concerns. Poland, on the other hand, has managed to reduce the contribution of households in financing healthcare by nearly 11 p.p. between 2000 and 2018, and the results of linear trend regression analysis confirm a decreasing trend over the years, with an average decrease of 0.5 per year. The observed improvement in Poland can be attributed to the growing importance of other financing sources, i.e., voluntary health insurance. Yet, the role of private insurance was rather negligible in the analyzed countries, and only in Slovenia did its share of healthcare spending reach 15% (data not presented).

The results of multiple regression analyses, where data for all countries were included (Table 3), indicated a small but statistically significant upward trend in current health expenditure as percentage of GDP and government health expenditure as percentage of general government spending. On the other hand, the public share in healthcare spending showed a downward trend, while no significant trend was found in the share of out-of-pocket payments. Moreover, the results confirm statistically significant differences in the four indicators of health expenditure across the EU-8 countries.

**Figure 1:**
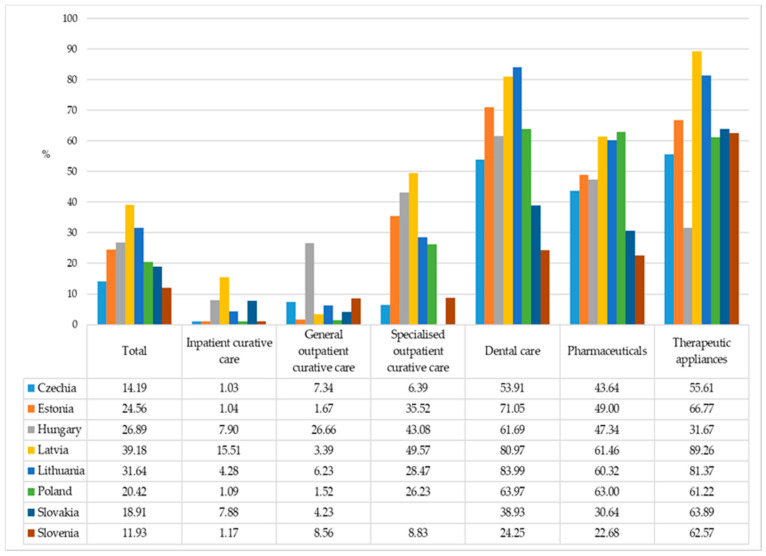
Household out-of-pocket payments as proportion of total health spending by type of service, 2018. Note: the percentages for total refer to share of household out-of-pocket payments in total current health expenditure.

Figure 1 shows that there is great diversity in the shares of out-of-pocket payments in financing different types of healthcare. Households mostly contribute to financing medical products (therapeutic appliances) (up to 89% in Latvia), dental care (up to 84% in Lithuania), and medicines (up to 63% in Poland). Nevertheless, the share of household out-of-pocket payments in financing outpatient specialized services is also high in some countries, e.g., 50% in Latvia and 43% in Hungary.

### 3.2. Healthcare Coverage

#### 3.2.1. Population Coverage

The results of our review indicate that the healthcare systems in the EU-8 countries are intended to serve the whole population, and the health insurance law requires all citizens and legally employed temporary residents to participate in the obligatory health insurance system, which is currently present in all countries but Latvia. Thus, the right to publicly financed healthcare is mainly conditional on the participation in the insurance scheme. Nevertheless, selected groups (usually those eligible for any kind of pension/social assistance, children, registered unemployed), which account for a significant share of population (>50% in Czechia, Estonia, Lithuania, Slovakia), either are insured by the state or are covered without contribution paid on their behalf.

The review of available evidence showed that the majority of analyzed countries have achieved universal (or near universal) population coverage for their respective benefit packages. Three countries (Czechia, Latvia, Slovenia) reported that 100% of the population is covered by the national health system. In another countries, the gaps in population coverage (up 9% in Poland) concern the citizens living and working abroad or are due to shortcomings of the insurance system, which is not flexible and transparent enough to permanently cover all individuals eligible for the coverage, e.g., those with nonstandard employment (part-time work, temporary employment, work based on employment relationship other than employment contracts, informal employment). More details are presented in Table 4.

#### 3.2.2. Service Coverage and Quality of Care

In the EU-8 countries, the benefit package is most often defined by positive lists (see Table 5), though in some countries negative lists, which explicitly exclude certain benefits from the coverage, are also present, i.e., Czechia, Hungary, and Latvia. In two countries (Lithuania, Slovenia), there are no comprehensive explicit lists of services included or excluded from the public coverage. In Czechia, although the list is defined, services not included on the list might be still provided free of charge to patients depending on their medical needs. The lists of services are revised on a rather ad hoc basis while the list of medicines is most often periodically updated (e.g., every two months in Poland). Decisions regarding benefits packages for medicines are also informed by Health Technology Assessment (HTA) and are more transparent, following EU regulations (Council Directive 89/105/EEC of 21 December 1988) [53]. The economic crisis of 2008 proved to have some impact on service coverage; e.g., in Estonia, Latvia, and Czechia, the benefit package has been restricted.

In the majority of the countries, service coverage is rather comprehensive, and includes prescribed spa treatment or even over-the-counter medicines if prescribed by a physician (Czechia), along with the basic healthcare services, such as primary healthcare, specialized inpatient and outpatient care, disease prevention, rehabilitation, prescription medicines, medical products, and emergency care. The services most often excluded from the benefit package are dental care for adults (with some services covered), employer-requested health examinations, medical certificates, and cosmetic surgery. Also, the scope of available medicines and medical products is considered limited in some countries. Nevertheless, rather small differences across the eight countries in the coverage of essential health services were confirmed by the data on the universal health coverage (UHC) service coverage index, which ranged from 71 in Latvia to 79 in Slovenia.

Although a relatively broad range of available services, patients might be faced with limits on the number of services to which they are entitled, e.g., infertility treatments in Hungary, rehabilitation and dental care in Poland. Access to care might be also restricted due to volume or quota limits imposed by public payers on healthcare providers to match the available public resources. Moreover, most of the countries do not specify any waiting time guarantees. This leads to long waiting times, which is one of the reasons for unmet needs and high out-of-pocket payments as a result of using care in the private sector.

The shortages of medical professionals and their uneven distribution might undermine timely and equal access to healthcare in the EU-8 countries. In 2018, on average across EU countries, there were 3.8 practicing doctors and 8.2 practicing nurses per 1000 population. Among the analyzed countries, the rates were similar or higher only for Czechia (for physicians and nurses), Estonia (for physicians), and Slovenia (for nurses).

The Health Consumer Index scores confirmed that quality of care in the countries at hand remains a challenge, as none of the analyzed countries reached the level of more than 750 points, which is a threshold for “green countries”, meaning those where patients positively assess the quality of healthcare services. The index scores were particularly low for Hungary, Poland and Latvia.

### 3.3. Patient Cost Sharing for Goods and Services in a Benefit Package

Obligatory patient cost sharing for outpatient services (GPs and/or specialists) and inpatient hospital services included in the benefit package is present in three countries (Estonia, Latvia, and Slovenia) (see Table 6). In these countries, the system of patient payments for services had already been introduced in the 1990s, and in later years only modified. In three countries, i.e., Czechia, Hungary, and Slovakia, cost sharing for healthcare services was implemented after 2000, but then withdrawn due to public and political opposition.

The countries included in the study rely strongly on cost sharing for medicines. In all countries, with the exemption of Czechia, patients need to pay for prescribed outpatient medicines in some form of percentage co-payment. A system of reference pricing is also in place, and patients additionally pay the cost above the reference price. In addition, medical products and dental care for adults, if included in the benefit package, are subject to heavy patient payments in all of the analyzed countries.

In all countries, cost sharing is accompanied by protection mechanisms, such as exemptions for vulnerable population groups or limits on payments. Yet, the extent of protection policy varies, and in some countries the mechanisms are considered weak (e.g., Latvia, Hungary, Poland). They do not always protect low-income individuals, or there is no overall cap on payments to relieve the financial burden for frequent users. In one country, i.e., Slovenia, patients are protected against payments by complementary private insurance that covers cost-sharing obligations.

## 4. Discussion

This paper presents the results of quantitative and qualitative data analysis on healthcare financing in eight CEE countries: Czechia, Estonia, Hungary, Latvia, Lithuania, Poland, Slovakia, and Slovenia. Based on available data, we have analyzed the changes in the level and structure of health spending over the last nearly two decades and the gaps in universal health coverage in these countries. The previous research on the topic has presented either a comparative perspective on health spending and universal health coverage e.g., [65,66,67] using quantitative data, or a specific aspect of healthcare financing (e.g., medicine reimbursement, e.g., [56,62], or patient cost sharing, e.g., [14,64]) or focused on individual countries (e.g., [49,63]). This study adds to the available literature by presenting a cross-country comprehensive analysis of healthcare financing and coverage using quantitative and qualitative data. Despite this strength of our study, the presented results should be interpreted, bearing in mind its limitations. As we have applied a desk research method, we might not have the most accurate and latest information on each of the analyzed countries. To some extent, we have mitigated this limitation by cross-checking data from different sources. The comparability of presented data (including quantitative data on healthcare expenditure) across the countries and over time is also limited, though we endeavored to use analogous sources of data for each country.

Our results show that in seven out of eight analyzed countries, the level of resources devoted to health in relation to country GDP has increased since 2000, and there was a statistically significant upward trend over time in most of the countries. Nevertheless, the rates can still be considered low when compared to the EU-27 average (8.3% in 2018) [54], placing the EU-8 countries among the lowest spenders in the EU. Thus, the distance between west and east, which reflects the diverse economic and political evolution of these two parts of Europe since the end of World War II [8,9], remains significant when it comes to healthcare expenditure.

The results also indicate that the EU-8 countries increase priority given to health when making decision concerning public spending. In the majority of the analyzed countries, the share of health spending in total government expenditure has risen since 2000, though a significant trend was evident only in a few countries, and the increase was rather small, allowing only Czechia to reach a level above the EU average [54]. Despite this positive change, government resources have not been sufficient to significantly improve healthcare financing structure, and overall, the contribution of public resources in healthcare financing in the EU-8 countries has shown a downward trend. This is due to reducing the government responsibility for healthcare financing in countries where, at the start (2000), healthcare funds came largely from public sources (Czechia, Estonia, Slovakia), and rather small or no progress in lowering out-of-pocket spending in other countries, where their level is still above the EU average of 22% [54].

The results of qualitative data analysis indicate that the EU-8 countries aimed to ensure universal population coverage of their public health systems, though in most of the EU-8 countries, a small fraction of the population remain uninsured. In fact, the numbers of uninsured individuals might be lower than indicated in the statistics, as some of the registered uninsured are living and working abroad and thus, are most likely covered by the systems in their countries of residence. Nevertheless, there is still a need to improve the transparency and flexibility of the systems to ensure stable coverage for all eligible individuals, including those in nonstandard employment. It is also worth noting that in the countries with a health insurance system, the entitlement to healthcare services for a significant part of the population (those who are economically inactive) is not conditional on paying insurance contributions. Hence, the insurance systems in these countries rely strongly on taxes to pay for noncontributing individuals. When the state does not take on the responsibility of contributing for the nonactive population (Estonia, Poland) or the contribution paid on their behalf is significantly lower than for the economically active population, the sustainability of healthcare financing might be at risk.

Our results also indicate that the low public health spending and the tension between needs and available resources have led to significant gaps in service and cost coverage as a result of the explicit decision to shift the cost of care to households, or implicit rationing.

Explicit mechanisms, such as excluding services and goods from the benefit package or introducing patient cost sharing, are commonly applied for medical products, dental care, and medicines. In the case of medicines, a transparent and systematic approach is often used as the list of covered pharmaceuticals is regularly updated based on HTA criteria. Nevertheless, such open decisions to reduce government responsibility for financing healthcare are politically challenging and might trigger public opposition. For these reasons explicit mechanisms are not always successfully applied in CEE countries when it comes to basic healthcare services (primary care, inpatient and outpatient specialist care). As our review shows, obligatory patient cost sharing for healthcare services is present in three countries (Estonia, Latvia, and Slovenia), while three other countries (Czechia, Hungary, and Slovakia) implemented and later withdrew patient payments due to public opposition. Previous evidence showed that public disapproval of obligatory payments for publicly financed healthcare services in CEE countries is mainly driven by dissatisfaction with quality and access to care, and the failure of the cost-sharing system to remedy this situation for patients [68].

Implicit measures that are not directly aimed at increasing out-of-pocket payments but might ultimately lead to patients paying for care, such as waiting times or volume limits imposed by public payers on healthcare providers, might be responsible for a significant part of out-of-pocket payments in the analyzed countries. This was confirmed by the relatively high share of out-of-pocket payments in financing outpatient specialist services observed in countries with comprehensive service and cost coverage (Hungary, Poland, and Lithuania). Long waiting times and poor quality of services might be the reasons for using privately financed healthcare services, purchasing over-the-counter medicines (commonly used in CEE countries as shown in the European Health Interview Survey [69], or even paying informally for care. The prevalence of informal patient payments has decreased over years in the CEE region, though they still constitute a barrier to access and a financial burden for households in many countries [63,70,71]. The 2017 Special Eurobarometer report on corruption, indicated that among the eight countries, informal patient payments (in cash or in kind) were most widespread in Hungary (reported by 17% of those who had used healthcare in the previous 12 months), while Slovenia and Estonia occupied the opposite end with 3% of users paying informally [72].

Another factor, apart from the availability of financial resources, that has been recognized as an important reason for limited access to health services and lengthening the average waiting time in CEE countries is the shortage of healthcare professionals [73,74,75]. The number of practicing doctors and nurses per 1000 population among EU-8 countries is significantly lower than the EU average. Moreover, between 2000 and 2018, the number of practicing doctors per capita increased in the vast majority of EU countries, while in the analyzed EU-8 countries this increase was marginal (especially slight changes were noticed in Poland, Latvia, Estonia, Hungary, and Slovakia) [54]. Research has also indicated that free health professional mobility disproportionally benefits richer countries from the “old” EU at the expense of less advantaged countries (including the EU-8 countries), which are not able to draw foreign-trained medical staff or retain their national health workforce [75].

Despite some common features of CEE countries, our results show a great diversity across the eight countries analyzed in this paper. This heterogeneity was observed during the transition period and has continued until recent years as a result of persisting economic, social, or political differences across the countries, which are indicated in Table 1 [8].

The greatest challenges are in Latvia, where low public resources spent on health do not allow for a broad scoped benefit package to be made available free of charge for patients. Hence, cost sharing is commonly applied and out-of-pocket payments are responsible for nearly 40% of current health expenditure in Latvia. The financial hardship for households is significant, with 13% of households experiencing catastrophic out-of-pocket payments, as shown by a recent WHO study [40]. Moreover, when compared to other European countries, Latvia is characterized by a high proportion of the population with unmet needs for medical examination or treatment (due to costs, distance, or waiting times), i.e., 6.2% in 2018, based on the EU-Statistics on Income and Living Conditions (EU-SILC) [76]. All this motivated the implementation of healthcare funding reform, which resulted in a shift from a tax-based to an insurance-based model. Nevertheless, the potential of such a change to solve Latvia’s health system problems has been disputed [45,77,78], and the reform has been recently cancelled in the fear of deepening inequalities in access to healthcare services.

Countries that stood out positively in our analysis were Czechia and Slovenia. Both countries are characterized by the highest spending on healthcare. Moreover, out-of-pocket payments in these countries constitute a relatively low share of healthcare expenditure, i.e., below 15%. In Czechia, this result was achieved through a high priority given to health and significant (though diminishing over the years) government involvement in covering healthcare costs (little use of cost sharing for publicly financed healthcare and broad coverage of services). In Slovenia, the government’s role in financing healthcare is smaller and patient cost sharing is commonly applied. However, voluntary private insurance, which is commonly purchased by Slovenians, is taking over the cost-sharing obligations, leaving households with no need to pay out-of-pocket.

The examples of Czechia and Slovenia present different ways of ensuring universal health coverage. On the one hand, by prioritizing budget and mobilizing more public resources for health, and on the other hand, by creating conditions for private insurance to take a more significant role in financing healthcare. The former measure is challenging in less wealthy countries where competition for scarce resources is greater, and calls for a strong political will. The latter however requires government capacity to control for and mitigate against the adverse effects of private insurance, such as risk selection or the inability of poor individuals and those with worse health status to purchase insurance. For this reason, it is not a commonly used option in European countries [79].

## 5. Conclusions

In the last two decades, the EU-8 countries have made moderate progress in achieving universal health coverage, with a still high reliance on out-of-pocket expenditure in many countries. The health and economic crisis caused by the recent COVID-19 pandemic will likely hinder countries’ efforts toward universal health coverage through, on the one hand, increasing health needs, and on the other hand, reducing public revenues and increasing people’s economic vulnerability.

This calls for a careful budget prioritization to secure additional healthcare resources to maintain a provision of essential healthcare services and protect households against financial catastrophe and impoverishment. This is also a time when new sources of revenues (external loans, grants) might play a more important role in financing healthcare. A revision of healthcare coverage might also be required, e.g., extending population coverage to ensure access to necessary healthcare services in order to control the pandemic, or reducing cost-sharing obligations for those most affected by the economic crisis.

Our results indicate that there is room for improving universal health coverage in CEE countries, extending the coverage to include all economically inactive groups, and improving the flexibility of the insurance system so that the continuity of insurance is maintained during labor market transitions. A systematic and evidence-based approach to establish a benefit basket and patient cost-sharing obligations might help to ensure more equal access to essential and efficient healthcare services. There is also a need for improving protection against cost-sharing obligations, particularly for those with a low ability to pay (through exemptions) and populations with higher needs (through caps on payments). Since gaps in the quality of care play an important role in burdening patients with out-of-pocket payments, they also call for greater policy attention.

This research does not remain without limitations, which are outlined in the discussion section. Yet, our results can serve as a base for further research on healthcare expenditure and health coverage in CEE countries. It is particularly relevant to study universal health coverage achievements, e.g., financial protection against catastrophic and impoverishing out-of-pocket payments, equity in access to healthcare, and quality of care. Studies on the effectiveness of policies and programs implemented in CEE countries to improve universal health coverage could also facilitate evidence-based policymaking. Finally, a more sophisticated analysis could be applied to study trends in healthcare expenditure, covering various determinants of healthcare expenditure, including demographic, economic, social, and health system factors.

## Figures and Tables

**Table 1:** Characteristics of the included countries.

	Czechia	Estonia	Hungary	Latvia	Lithuania	Poland	Slovakia	Slovenia
Population (thousands) (2018)	10,610	1319	9778	1934	2809	37,977	5443	2067
Population density (persons per km^2^) (2018)	137.7	30.4	107.1	30.4	44.7	123.6	111.8	102.9
Life expectancy at birth, total (years) (2018)	79.1	78.5	76.2	75.1	76.0	77.7	77.4	81.5
Old-age dependency ratio (2018)	29.6	30.6	28.5	31.4	30.1	25.3	22.5	29.6
GDP per capita, PPP (current international $) (2018)	41,036	36,222	32,086	30,736	36,011	31,851	32,538	38,841
Human Development Index ^†^ (2018)	0.898	0.889	0.850	0.863	0.876	0.877	0.858	0.912
Government Effectiveness Index ^‡^ (2018)	0.92	1.19	0.49	1.04	1.07	0.66	0.71	1.13
Healthcare for the sick: percentage claiming it should be the government’s responsibility (2016) ^§^	95.8	n.d.	98.0	98.0	96.0	n.d.	98.2	98.9

^†^ The Human Development Index (HDI) is a composite index that captures human development in three dimensions: health (measured by life expectancy at birth), education (measured by mean of years of schooling for adults aged 25 years and more, and expected years of schooling for children of school entering age), and income (measured by gross national income per capita) [30]. ^‡^ Government Effectiveness Index is the World Bank’s aggregate governance indicator that reflects perceptions of the quality of public services, the quality of the civil service and the degree of its independence from political pressures, the quality of policy formulation and implementation, and the credibility of the government’s commitment to such policies. Estimates range from approximately −2.5 (weak) to 2.5 (strong) governance performance [31]. ^§^ Results of a cross-sectional survey on the nationally representative sample of all individuals aged over 15 years. Survey question: “On the whole, do you think it should or should not be the government’s responsibility to provide healthcare for the sick. Answers “definitely should” and “probably should” are included [32].

**Table 2:** Healthcare expenditure in the EU-8 countries (Czechia, Estonia, Hungary, Latvia, Lithuania, Poland, Slovakia, and Slovenia) and the results of the simple regression analysis.

	2000	2018	Min. Annual Growth Rate (Year)	Max. Annual Growth Rate (Year)	Regression Model
	**Current health expenditure as % of GDP**				
Czechia	5.72	7.65	−5.4 (2015)	14.7 (2009)	y = 0.098x + 5.790, R^2^ = 0.758, *p* < 0.01
Estonia	5.16	6.66	−8.0 (2011)	14.6 (2008)	y = 0.111x + 4.553, R^2^ = 0.784, *p* < 0.01
Hungary	6.78	6.70	−7.3 (2007)	14.0 (2003)	y = −0.027x + 7.542, R^2^ = 0.129, *p* = 0.131
Latvia	5.45	6.21	−9.3 (2011)	13.4 (2004)	y = 0.011x + 5.661, R^2^ = 0.046, *p* = 0.376
Lithuania	6.19	6.57	−10.9 (2004)	17.0 (2009)	y = 0.037x + 5.891, R^2^ = 0.233, *p* < 0.05
Poland	5.30	6.33	−3.2 (2018)	8.5 (2008)	y = 0.050x + 5.643, R^2^ = 0.644, *p* < 0.01
Slovakia	5.30	6.69	−8.2 (2014)	18.7 (2004)	y = 0.090x + 5.828, R^2^ = 0.395, *p* < 0.01
Slovenia	7.80	8.33	−4.0 (2007)	8.7 (2009)	y = 0.043x + 7.787, R^2^ = 0.439, *p* < 0.01
	**Domestic general government health expenditure as % of general government expenditure**				
Czechia	12.38	15.54	−4.6% (2003)	15.8 (2013)	y = 0.195x + 11.508, R^2^ = 0.824, *p* < 0.01
Estonia	10.80	12.54	−6.6 (2002)	5.6 (2003)	y = 0.119x + 10.191, R^2^ = 0.851, *p* < 0.01
Hungary	9.86	9.92	−8.1 (2007)	19.7 (2003)	y = −0.045x + 10.523, R^2^ = 0.154, *p* = 0.097
Latvia	7.43	9.60	−9.2 (2008)	23.7 (2004)	y = 0.037x + 8.468, R^2^ = 0.083, *p* = 0.232
Lithuania	10.57	12.70	−25.9 (2004)	11.8 (2002)	y = 0.067x + 10.947, R^2^ = 0.141, *p* = 0.113
Poland	8.59	10.83	−5.2 (2010)	8.1 (2008)	y = 0.132x + 8.561, R^2^ = 0.872, *p* < 0.01
Slovakia	8.89	12.65	−7.6 (2015)	17.0 (2001)	y = 0.129x + 11.112, R^2^ = 0.336, *p* < 0.01
Slovenia	11.72	13.80	−18.5 (2013)	15.7 (2014)	y = 0.053x + 11.774, R^2^ = 0.157, *p* = 0.093
	**Government/compulsory scheme expenditure as % of current health expenditure**				
Czechia	89.80	83.03	−3.1 (2008)	1.4 (2009)	y = –0.472x + 89.835, R^2^ = 0.801, *p* < 0.01
Estonia	76.97	73.67	−2.7 (2017)	1.8 (2001)	y = −0.122x + 77.231, R^2^ = 0.312, *p* < 0.05
Hungary	69.65	69.45	−2.7 (2007)	2.0 (2002)	y = −0.129x + 69.903, R^2^ = 0.224, *p* < 0.05
Latvia	50.75	59.88	−5.0 (2012)	12.7 (2004)	y = 0.499x + 52.309, R^2^ = 0.393, *p* < 0.01
Lithuania	68.51	67.05	−11.7 (2004)	5.2 (2007)	y = −0.272x + 71.969, R^2^ = 0.280, *p* < 0.05
Poland	68.88	71.49	−2.0 (2004)	3.1 (2001)	y = 0.059x + 69.543, R^2^ = 0.085, *p* = 0.226
Slovakia	89.16	80.13	−11.6 (2004)	8.7 (2008)	y = −0.425x + 82.591, R^2^ = 0.135, *p* = 0.121
Slovenia	72.90	72.93	−2.2 (2007)	2.8 (2008)	y = −0.043x + 73.042, R^2^ = 0.083, *p* = 0.231
	**Out-of-pocket health expenditure as % of current health expenditure**				
Czechia	10.20	14.19	−11.2 (2013)	18.4 (2008)	y = 0.300x + 10.242, R^2^ = 0.629, *p* < 0.01
Estonia	20.37	24.56	−8.5 (2008)	9.4 (2017)	y = 0.186x + 20.127, R^2^ = 0.516, *p* < 0.01
Hungary	27.33	26.89	−4.5 (2002)	5.4 (2001)	y = 0.053x + 26.637, R^2^ = 0.068, *p* = 0.280
Latvia	47.66	39.18	−16.3 (2004)	10.3 (2012)	y = −0.404x + 44.834, R^2^ = 0.258, *p* < 0.05
Lithuania	27.15	31.64	−10.8 (2007)	36.1 (2004)	y = 0.291x + 26.873, R^2^ = 0.338, *p* < 0.01
Poland	31.12	20.42	−10.5% (2018)	6.8 (2004)	y = −0.457x + 29.897, R^2^ = 0.867, *p* < 0.01
Slovakia	10.84	18.91	−23.2 (2008)	71.2 (2004)	y = 0.335x + 16.230, R^2^ = 0.134, *p* = 0.123
Slovenia *	12.47	11.93	−7.3 (2008)	11.1 (2007)	y = −0.033x + 12.905, R^2^ = 0.133, *p* = 0.165

* Due to the lack of data, the value of the indicator, out-of-pocket health expenditure as % of current health expenditure, for Slovenia is from 2003, and the regression analysis covers data for 2003–2018.

**Table 3:** The results of the multiple regression analysis.

	Current Health Expenditure as % of GDP	Domestic General Government Health Expenditure as % of General Government Expenditure	Government/Compulsory Scheme Expenditure as % of Current Health Expenditure	Out-of-Pocket Health Expenditure as % of Current Health Expenditure
	Coeff. (S.E.)	Coeff. (S.E.)	Coeff. (S.E.)	Coeff. (S.E.)
Time	0.052 *** (0.007)	0.086 *** (0.012)	−0.113 * (0.048)	0.037 (0.046)
Czechia	1.105 *** (0.145)	2.080 *** (0.254)	9.109 *** (1.043)	−8.743 *** (0.973)
Estonia	ref.	ref.	ref.	ref.
Hungary	1.607 *** (0.145)	−1.307 *** (0.254)	−7.394 *** (1.043)	5.185 *** (0.963)
Latvia	0.109 (0.145)	−2.541 *** (0.254)	−18.716 *** (1.043)	18.812 *** (0.963)
Lithuania	0.599 *** (0.145)	0.234 (0.254)	−6.7574 *** (1.043)	7.798 *** (0.963)
Poland	0.472 ** (0.145)	−1.497 ***(0.254)	−5.879 *** (1.043)	3.349 *** (0.963)
Slovakia	1.064 *** (0.145)	1.024 *** (0.254)	2.329 * (1.043)	−2.399 * (0.963)
Slovenia	2.547 *** (0.145)	0.926 *** (0.254)	−3.396 ** (1.043)	−9.457 *** (0.963)
Constant	5.149 *** (0.122)	10.521 *** (0.213)	77.141 *** (0.878)	21.608 *** (0.825)
Observations	152	152	152	149
F test	F(8, 143) = 66.82 ***	F(8, 143) = 71.23 ***	F(8, 143) = 108.1 ***	F(8, 140) = 161.5 ***
R-squared	0.789	0.799	0.858	0.897
Adj. R-squared	0.777	0.788	0.850	0.891

*** *p* < 0.001, ** *p* < 0.01, * *p* < 0.05.

**Table 4:** Population coverage in the EU-8.

Country	Basis for Entitlement	% of Population Covered	Noncovered Groups	Contribution Paid by the Government or Coverage without Contribution Paid on Behalf of Noncontributors	Additional Information
Czechia	Participation in insurance scheme [35].	100% [36,37].	n/a	State-paid contribution for 17 population groups (58% of population), including children and students up to the age of 26, disabled people, pensioners, the unemployed, and informal caregivers [22,38].	The contribution for state-insured groups is fixed and substantially lower than an average contribution from salary [22].
Estonia	Participation in insurance scheme [39].	94–95% [36,39,40].	Individuals working abroad and those inactive/unemployed or with nonstandard employment [23,40,41].	State-paid contribution for 24% of the insured (e.g., pensioners, people on parental leave, caregivers). No contribution is paid for ~26% of the covered population (e.g., children, students up to the age of 24, the unemployed) [23,39,40].	The government started paying contributions for pensioners in 2018 in order to increase the financial stability of the system [23,39]. Health coverage has been gradually extended, e.g., registered jobseekers were included in 2007 [39,40].
Hungary	Participation in insurance scheme [24,42].	94–95% [24,36,42,43].	Individuals working abroad or without a fixed address [24].	State-paid contribution for selected groups, mainly pensioners, minors, students [42].	The government pays for a significant share of the health insurance fund exceeding revenues from wage-based health insurance contributions [43]. In practice, noncovered individuals also receive necessary healthcare services [24,42].
Latvia	Residence [25].	100% [25,36,44].	n/a	n/a	Between 2018 and 2019, a mandatory health insurance system was in place. The full benefit package was available for individuals paying the insurance contribution and to those in one of 21 population groups covered by the state (e.g., children, pensioners). Others were granted access to a minimum benefit package financed by the state (emergency care, primary care, maternity care, psychiatric care, treatment of infectious diseases, and reimbursement of pharmaceuticals) [25,45].
Lithuania	Participation in insurance scheme [46].	98% [26,36].	Individuals not in regular employment, people working abroad [26].	State-paid contribution for 55–60% of the population, including pensioners, social assistance beneficiaries, children under 18, students, registered unemployed, disabled individuals, patients suffering from certain communicable diseases [46,47].	Emergency healthcare is provided free of charge to all permanent residents irrespective of their insurance status [26,47].
Poland	Participation in insurance scheme [48,49].	91–93% [27,36,48,49].	Individual working abroad, nonregistered family members, nonregistered homeless or unemployed people [27,49].	State-paid contribution for farmers of small farms, registered jobseekers, recipients of income support, clergy, children, and students if they are not co-insured family members. No contribution is paid for co-insured family members (22% of the insured population) [48,49].	For pregnant women and children under 18, the right to publicly financed healthcare can be granted irrespective of their insurance status. Since 2017, all people, regardless of insurance status, have had free access to primary care. Individuals not insured but eligible for public coverage can be insured retroactively avoiding out-of-pocket payments [27,48,49].
Slovakia	Participation in insurance scheme [28,50].	94–96% [28,36,50].	Mostly individuals working abroad [28,50].	State-paid contribution for the economically inactive (dependent family members, students and pensioners) (in total >50% of population) [28,50].	The insurance rate of the state-paid contribution has been fluctuating over the years, and now it is lower than the rate for the economically active, contributing to a financial deficit in healthcare system [51].
Slovenia	Participation in insurance scheme [29,52].	99.5–100% [29,36,52].	Ethnic minorities, undocumented migrants, and nonregistered homeless people [29,36,52].	State-paid contribution for pensioners, the unemployed, individuals without income, prisoners, and war veterans [52].	In addition to public coverage, about 95% of the population purchase voluntary health insurance to cover obligatory patient payments [29].

Note: data from OECD Report [36] on the share of population covered refer to population coverage for a core set of services (which generally include consultations with physicians, tests/examinations and hospital care) under public programs and through primary private health insurance. Yet, the scale of private insurance as a primary source of coverage is negligible in the analyzed countries. n/a—not applicable.

**Table 5:** Service coverage and quality of care in the EU-8 countries.

Country	Benefit Package	Example of Included/Excluded Services	Quality and Access to Healthcare	Changes in the Benefit Package	Additional Information ^†,‡^
Czechia	Positive list of services, medicines, medical products. Negative list of services explicitly excluded [37].	Included: in vitro fertilization, spa treatments, over-the-counter medicines (if prescribed), dental care (least expensive options) [22,37]. Excluded: cosmetic surgery, dental treatments, voluntary abortions, employer-requested health examinations, medical certificates, some medical aids, e.g., prescription glasses [22,37].	There might be limitations on volume of services provided by specific providers [37]. No problems with waiting times [33]. Regional disparities in the distribution of physicians [22] 4.0 doctors and 8.1 nurses per 1000 population [54].	During the economic crisis of 2008, dental benefits were restricted [55]. Periodic (several times a year) amendments of the medicine list [56].	Services not included on the positive list may still be reimbursed, depending on the needs of individual patients. Also, there are exceptional cases in which items on the negative list may be reimbursed [37]. UHC service coverage index: 76 [57]. HCI: 731 points (14th place) [57].
Estonia	Positive lists of services, medicines, medical products [39].	Included: dental care for children, most essential dental services for adults [23,39,40]. Excluded: cosmetic surgery, alternative therapies, optician services [39,40].	Maximum waiting time guarantees, however often not met for specialist services [23,40]. Medical professional shortages, particularly nurses [23]. 3.0 doctors and 6.3 nurses per 1000 population [54].	Between 2002 and 2017, cash benefit instead of in-kind dental care benefits for adults). Coverage of dental care for adults restricted during the economic crisis of 2008, restored in 2017 [23,39]. Benefit package updated at least once a year [23].	UHC service coverage index: 75 [58]. HCI: 729 points (15th place) [57].
Hungary	Negative list of services. Positive and negative list of medicines [42,57].	Included: spa treatment, infertility treatments (limits on number of benefits) [42]. Excluded: cosmetic surgery, medical certificates, abortion or sterilization without medical indication, selected dental services for adults [42].	Performance volume limit set for each provider [24]. Long waiting times [42]. Shortages and uneven distribution of medical professionals [24]. 3.4 doctors and 6.6 nurses per 1000 population [54].	Ad hoc revision of medicine lists [56].	UHC service coverage index: 74 [58]. HCI: 565 points (33rd place) [57].
Latvia	Positive list of medicines and certain services. Negative lists of services explicitly excluded [25].	Included: dental care for children [39] Excluded: dental care for adults, some rehabilitation services, employer-requested health examinations, sight correction, hearing aids for adults, psychotherapy, spa treatment, abortion (if no medical or social indications) [44,45].	Volume limits on contracted services. Lack of waiting time guarantees and long waiting times [25,44,45]. Shortages and uneven geographical distribution of medical professionals [25]. 3.3 doctors and 4.4 nurses per 1000 population [54].	Reduction of benefit package after the economic crisis in 2008 [45]. Periodic (every 3 months) amendments of the medicine list [56].	The scope of the benefit package is considered relatively limited, particularly for outpatient care, medicines, medical devices [25,45]. UHC service coverage index: 71 [58]. HCI: 605 points (30th place) [57].
Lithuania	No explicitly defined list of services. Positive list of medicines and medical products [26,46].	Included: dental care for children and individuals on income support, in vitro fertilization (since 2017) [26,46,47]. Excluded: dental care for adults, medical certificates, nonmedical cosmetics, over-the counter medicines, occupational health check-ups, abortions, substance abuse treatment [26,46,47].	Long waiting times [46]. Uneven geographical distribution of medical professionals; shortages of nurses [26]. 4.6 doctors and 7.8 nurses per 1000 population [54].	Periodic revision of medicine list since 2019 [26].	Coverage of pharmaceuticals and medical products is limited [26,46]. UHC service coverage index: 73 [58]. HCI: 622 points (28th place) [57].
Poland	Positive lists for services, medicines, medical products [48,49].	Included: spa treatment, basic dental services for adults with more services for children [48,49]. Excluded: most dental care for adults, medical certificates and nonmedical cosmetics, over-the counter medicines, in vitro fertilization [48,49].	Limits on the volume of services contracted. Lack of waiting time guarantees and long waiting times [27,49]. Sever shortages of medical professionals and their uneven distribution [27]. 2.4 doctors and 5.1 nurses per 1000 population [54].	Periodic (every 2 months) amendments of the medicine list [56]. Ad hoc revision of the service list. In vitro fertilization was covered between 2013 and 2016, and then was excluded due to political reasons [48].	Lists of guaranteed services in primary care, outpatient specialist care, and hospital care are fairly comprehensive. In the case of some services (rehabilitation, dental care, medical products), limits on the number of services to be provided per person [27,49]. UHC service coverage index: 75 [58]. HCI: 585 points (32nd place) [57].
Slovakia	Positive list of services, medical products and medicines [50].	Included: spa treatment, some dental services for adults [50]. Excluded: most dental care, patient-requested anesthesia, paternity tests, specialist visits without referral, treatment caused by substance abuse, cosmetic plastic surgery, abortion upon request of the patient, sterilization [50,59].	Budget ceilings resulting in waiting times for specialists [59]. Shortages of healthcare professionals and their uneven distribution [28]. 3.5 doctors and 5.7 nurses per 1000 population [54].	Periodic (every 3 months) amendments of the medicine list [56].	The benefit package is broadly defined. Some attempts to define a narrower benefit package (2002–2004) were unsuccessful [59]. UHC service coverage index: 77 [58]. HCI: 722 points (17th place) [57].
Slovenia	The list of covered services only broadly defined [52,60]. Positive and negative list of medicines [52].	Included: dental care for children and adults, contraception, infertility treatment, artificial insemination, sterilization, abortion, costs of travel to health facilities. Excluded: cosmetic surgery [52].	Maximum waiting times, but not always met for specialist care [29,52]. Low number of physicians [29]. 3.2 doctors and 10.1 nurses per 1000 population [54].		Although the majority of services are covered, they might be subject to percentage co-payments (up to 90%) (see: section on patient cost sharing). UHC service coverage index: 79 [58]. HCI: 678 points (21st place) [57].

^†^ UHC—universal health coverage. UHC service coverage index is Sustainable Development Goal (SDG) indicator. It is defined as a coverage index for essential health services based on tracer interventions that include reproductive, maternal, newborn and child health, infectious diseases, noncommunicable diseases, and service capacity and access. The index ranges from 0 to 100 [58]. ^‡^ HCI—Health Consumer Index. HCI presents the assessment of the performance of national healthcare systems in 35 countries (on the 1000-point scale) using 46 indicators, from the areas such as patient rights and information, access to care, treatment outcomes, range and reach of services, prevention and use of pharmaceuticals [57].

**Table 6:** Patient cost sharing for healthcare in the EU-8 countries.

Country	Cost Sharing for Outpatient Services (GPs and/or specialists) and Inpatient Hospital Services	Cost Sharing for Outpatient Medicines	Cost Sharing for other Goods and Services	Protection Mechanisms (Co-Payment Limits, Exemptions)	Additional Information
Czechia	None (except for ambulatory services outside standard office hours) [22,37,61].	The difference between the price and the reimbursement amount [22].	Payments for medical products and dental care beyond the standard package (cost above the reimbursement amounts) [22,37,61].	A yearly payment limit of 5000 CZK (~€200) for payments for medicines. Lower limits apply to children up to 18 years of age and seniors (both since 2018), as well as disabled people (since 2020). Selected vulnerable groups are fully exempt [22,37].	Between 2008 and 2015, there were co-payments for services: 30 CZK (€1.20) per doctor visit, 60 CZK (€2.40) (100 CZK since 2011) per hospital day. There was also cost sharing for medicines in the form of a flat fee of 30 CZK (€1.20) for each prescribed pharmaceutical (since 2012 per prescription). Due to the unpopularity of user fees, some local governments (controlled by the opposition) had reimbursed patients for the user charges before fees were fully abolished in 2015 [37,61].
Estonia	Co-payments for outpatient specialist care (€5 per visit), inpatient care (€2.50 per day), and primary care home visits (€5 per visit) (otherwise primary care free-of-charge) [39,40].	Co-payment for each prescription (€2.50), a percentage co-payment (0%, 25%, 50%), and any additional costs above the reference price [39,40].	Dental care for adults: percentage co-payment of 50% with a benefit cap of €40 per year (15% and €85 respectively for some vulnerable groups) For medical products: percentage co-payments of 10% or 50% [39].	A cap on payments for hospital stays (€25 per hospitalization). Caps on payments for outpatient medicines, i.e., if the total spending on prescription medications in a year reaches €100, then the insurance fund starts to reimburse 50% of the cost above €100. This reimbursement rate increases up to 90% for out-of-pocket spending above €300. Over the years, these spending thresholds have been decreasing [23,39,40]. Exemption or fee reduction for selected vulnerable groups (children, pregnant women, pensioners) [40].	Cost sharing for services was first introduced in 1995. The system was modified by the 2002 Health Insurance Act, which set the maximum co-payment levels. In 2013, the maximum fee level was increased. Cost sharing for dental care was introduced in 2017, when in-kind dental care was included in the benefit package [39].
Hungary	None [42].	Percentage co-payments (up to 75%) or a fixed co-payment. The co-payment rate depends on the therapeutic value of the medicine, the severity and status of the disease (with lower rates for more severe or longer lasting disease) [24,56,62].	Different flat and percentage co-payment rates for medical products. Cost sharing for dental care (adults) [42].	A monthly personal budget of up to 12,000 HUF (€40) to cover co-payments for prescribed medicines for specific vulnerable groups (the disabled, low-income individuals). Limited exemption scheme for some vulnerable population groups [24].	Between 2007 and 2008, patients paid a flat co-payment of 300 HUF (approx. €1) per outpatient visit and a per diem for inpatient care (for max. 20 visits/days of hospitalization per year). The fees were withdrawn as a consequence of a nation-wide referendum [14,63].
Latvia	Co-payment of €1.42 per primary care visit, €4.28 per specialist visit, and €10 per day of hospitalization [44,45].	A flat fee of €0.71 per prescription or percentage co-payments of 25% or 50%, and any additional costs above the reference price [44,45].	For medical products: a flat fee of €0.71 per prescription or percentage co-payments of 25% or 50%, and any additional costs above the reference price [44,45].	Caps on the total annual payments for inpatient and outpatient services (€569 per person per year) and on payments for inpatient stay (€356 per hospitalization) (but no cap on payments for medicines and medical products). Exceptions from payments for some population groups, including children, pregnant women, people suffering from certain diseases, and since 2009, for low-income individuals [44,45].	Flat co-payments were first implemented in 1995, and in the late 90s, a percentage co-payment (up to 25% of service costs) was applied to selected services. Due to the complexity of the system, in 2004, it was simplified with only flat co-payments remaining [64]. In 2009, due to fiscal pressure, patient payments increased substantially, resulting in weakened financial protection. Thus, in the subsequent years, some fee reduction took place [45].
Lithuania	None (except for visits to specialists without referrals) [46,47]	Patients pay the full price for medicines unless they fall into certain vulnerable groups (children, pensioners, the disabled, low-income individuals), for which full or partial reimbursement is applied (list B of medicines), or suffer from certain diseases, e.g., tuberculosis, cancers, schizophrenia, metabolic diseases, or asthma, for which medicines are now fully reimbursed (list A of medicines). Everyone pays the costs above the reference price [26,47,56].	None The majority of population pay the full price for medical products and dental care [46,47].	A 50% reimbursement rate for medicines on list B for pensioners, partially disabled and recipients of social benefits. Full reimbursement is available for children, the severely disabled, people with specific conditions, and since 2020, for low-income older adults [26,47,56].	Providers might charge patients the difference between the price and the actual cost of a treatment if patients opt for a treatment that is more expensive. There are concerns regarding the poor regulation and lack of transparency in these charges [46,47]. Since 2017, there have been significant changes in the reimbursement policy aimed at decreasing out-of-pocket payments for medicines. Before April 2019, there was percentage co-payment (50%, 20%, or 10%) for medicines on a disease-specific list (list A) [26].
Poland	None [48,49].	Fixed co-payment of PLN 3.20 (€0.7) or percentage co-payments of 30% or 50%, and any cost above the reference price [48,56].	For medical products: payments on top of the reimbursement price if patients opt for more expensive products, and percentage co-payments for some products. No cost sharing for dental care. The majority of the population pay the full price for dental care [48,49].	Exemptions apply to only a few groups, i.e., veterans, organ or blood donors, people aged 75+ (since 2016, for a broad range of medicines), pregnant women (since 2020, for certain medicines) children (only for some medical products) [48,49].	Until 2010, extra billing in dental care was allowed, i.e., patients had had the possibility of opting for more expensive materials within the publicly financed system and pay any extra cost [49].
Slovakia	None [50].	Different percentage co-payment rates (0–100%) Many medicines (1/3) provided free of charge. Flat fee for prescription €0.17 [50,56].	Different percentage co-insurance rates for medical products. Cost sharing for some dental services [50].	Maximum limits for co-payments for prescribed medicines. A wide range of medical devices with individually reduced cost sharing [50].	Between 2003 and 2006, co-payments for physician consultation, prescriptions (approximately €0.7), hospitalization (approximately €1.7 per day), and emergency care visits (approximately €2) were in place. In 2006, co-payments for outpatient and inpatient care were abolished, while other co-payments were reduced. Yet, providers continued to collect payments for health and health-related services. In 2015, a regulation was introduced banning such practices [50,64].
Slovenia	Percentage co-payments from 10% to 90% depending on the type of services [52].	Percentage co-payments (max. 30% for medicines on the positive list and min. 75% for medicines on the intermediate list) [52].	Percentage co-payments for dental care and medical products	Exempt from cost sharing are: children, students, unemployed individuals, low-income individuals, pregnant women, and chronically ill people [52,60].	Cost sharing was introduced in 1992 along with the compulsory health insurance system [14]. A vast majority of the population has private complementary insurance to cover cost-sharing obligations [52,60]. The economic crisis of 2008 led to an increase in co-payments [29].

## Data Availability

The data presented in this study are openly available in OECD Health Statistics Online Database, and WHO Global Health Expenditure Database, reference number [33,34].

## Supplementary Materials

The following are available online at https://www.mdpi.com/1660-4601/18/4/1382/s1, Figure S1: Current health expenditure as % of GDP in Czechia, 2000–2018, Figure S2: Current health expenditure as % of GDP in Estonia, 2000–2018, Figure S3: Current health expenditure as % of GDP in Hungary, 2000–2018, Figure S4: Current health expenditure as % of GDP in Latvia, 2000–2018, Figure S5: Current health expenditure as % of GDP in Lithuania, 2000–2018, Figure S6: Current health expenditure as % of GDP in Poland, 2000–2018, Figure S7: Current health expenditure as % of GDP in Slovakia, 2000–2018, Figure S8: Current health expenditure as % of GDP in Slovenia, 2000–2018, Figure S9: Domestic general government health expenditure as % of general government expenditure in Czechia, 2000–2018, Figure S10: Domestic general government health expenditure as % of general government expenditure in Estonia, 2000–2018, Figure S11: Domestic general government health expenditure as % of general government expenditure in Hungary, 2000–2018, Figure S12: Domestic general government health expenditure as % of general government expenditure in Latvia, 2000–2018, Figure S13: Domestic general government health expenditure as % of general government expenditure in Lithuania, 2000–2018, Figure S14: Domestic general government health expenditure as % of general government expenditure in Poland, 2000–2018, Figure S15: Domestic general government health expenditure as % of general government expenditure in Slovakia, 2000–2018, Figure S16: Domestic general government health expenditure as % of general government expenditure in Slovenia, 2000–2018, Figure S17: Government/compulsory scheme expenditure as % of current health expenditure in Czechia, 2000–2018, Figure S18: Government/compulsory scheme expenditure as % of current health expenditure in Estonia, 2000–2018, Figure S19: Government/compulsory scheme expenditure as % of current health expenditure in Hungary, 2000–2018, Figure S20: Government/compulsory scheme expenditure as % of current health expenditure in Latvia, 2000–2018, Figure S201 Government/compulsory scheme expenditure as % of current health expenditure in Lithuania, 2000–2018, Figure S22: Government/compulsory scheme expenditure as % of current health expenditure in Poland, 2000–2018, Figure S23: Government/compulsory scheme expenditure as % of current health expenditure in Slovakia, 2000–2018, Figure S24: Government/compulsory scheme expenditure as % of current health expenditure in Slovenia, 2000–2018, Figure S25: Out-of-pocket health expenditure as % of current health expenditure in Czechia, 2000–2018, Figure S26: Out-of-pocket health expenditure as % of current health expenditure in Estonia, 2000–2018, Figure S27: Out-of-pocket health expenditure as % of current health expenditure in Hungary, 2000–2018, Figure S28: Out-of-pocket health expenditure as % of current health expenditure in Latvia, 2000–2018, Figure S29: Out-of-pocket health expenditure as % of current health expenditure in Lithuania, 2000–2018, Figure S30: Out-of-pocket health expenditure as % of current health expenditure in Poland, 2000–2018, Figure S31: Out-of-pocket health expenditure as % of current health expenditure in Slovakia, 2000–2018, Figure S32: Out-of-pocket health expenditure as % of current health expenditure in Slovenia, 2000–2018, Table S1: Health care expenditure in the EU-8 countries, 2000–2018.
