# Supplementary material for: Financing Healthcare in Central and Eastern European Countries: How Far Are We from Universal Health Coverage?
Source: Int J Environ Res Public Health. 2021 Feb 3;18(4):1382. doi: 10.3390/ijerph18041382 (PMC7913209; doi:10.3390/ijerph18041382)

Table S1: Health care expenditure in the EU-8 countries, 2000-2018.

	Czechia	Estonia	Hungary	Latvia	Lithuania	Poland	Slovakia	Slovenia
**Current health expenditure as % of GDP**								
2000	5.72	5.16	6.78	5.45	6.19	5.30	5.30	7.80
2001	5.89	4.78	6.83	5.79	6.01	5.68	5.33	7.88
2002	6.20	4.70	7.12	5.78	6.14	6.08	5.51	8.02
2003	6.55	4.86	8.12	5.50	6.18	5.96	5.45	8.10
2004	6.39	5.12	7.77	6.24	5.51	5.87	6.47	7.94
2005	6.38	5.06	8.00	5.87	5.64	5.81	6.59	8.00
2006	6.20	4.77	7.79	5.74	5.84	5.80	6.85	7.82
2007	6.02	4.98	7.22	5.54	5.76	5.87	7.18	7.51
2008	6.36	5.71	7.10	5.62	6.29	6.37	6.96	7.86
2009	7.29	6.51	7.24	6.14	7.36	6.59	7.95	8.54
2010	6.93	6.27	7.51	6.14	6.83	6.42	7.74	8.57
2011	6.98	5.77	7.53	5.57	6.51	6.23	7.36	8.54
2012	7.03	5.79	7.45	5.41	6.29	6.20	7.55	8.72
2013	7.81	5.98	7.25	5.40	6.14	6.38	7.51	8.74
2014	7.65	6.08	7.07	5.46	6.20	6.25	6.89	8.50
2015	7.24	6.35	6.89	5.69	6.50	6.40	6.79	8.52
2016	7.15	6.50	7.05	6.21	6.64	6.54	6.99	8.49
2017	7.23	6.62	6.79	6.01	6.47	6.54	6.77	8.19
2018	7.65	6.66	6.70	6.21	6.57	6.33	6.69	8.33
**Domestic general government health expenditure as % of general government expenditure**								
2000	12.38	10.80	9.86	7.43	10.57	8.59	8.89	11.72
2001	11.92	10.70	9.66	8.11	11.37	8.87	10.40	11.41
2002	12.18	9.99	9.67	8.16	12.71	9.37	10.70	12.29
2003	11.62	10.56	11.57	8.17	13.68	8.93	11.75	12.36
2004	13.14	11.11	11.09	10.10	10.13	9.08	13.12	12.26
2005	12.86	11.10	11.36	9.55	10.05	9.00	12.33	12.45
2006	12.81	10.65	10.66	9.80	10.62	9.01	12.20	12.32
2007	12.49	11.09	9.79	9.89	10.75	9.54	13.50	12.15
2008	12.68	11.05	9.85	8.98	11.67	10.31	14.07	12.58
2009	13.68	11.02	9.71	8.24	11.78	10.54	13.08	12.46
2010	13.24	11.65	10.17	8.15	11.43	10.00	13.13	12.35
2011	13.57	11.66	10.10	8.77	10.73	10.09	13.01	12.13
2012	13.20	11.32	9.79	8.62	11.57	10.10	13.21	12.51
2013	15.28	11.82	9.57	8.59	11.32	10.52	13.08	10.20
2014	14.89	12.22	9.39	8.57	11.88	10.48	12.68	11.79
2015	14.28	12.19	9.22	8.79	12.22	10.64	11.71	12.45
2016	14.83	12.42	10.11	9.40	12.77	10.95	13.29	13.26
2017	15.18	12.36	9.93	9.03	12.72	10.96	12.90	13.34
2018	15.54	12.54	9.92	9.60	12.70	10.83	12.65	13.80
**Government/compulsory scheme expenditure as % of current health expenditure**								
2000	89.80	76.97	69.65	50.75	68.51	68.88	89.16	72.90
2001	89.36	78.36	68.17	48.73	71.57	71.02	89.09	71.47
2002	90.00	76.56	69.57	49.58	73.93	70.44	88.98	73.37
2003	89.39	76.66	70.54	49.83	75.38	69.16	87.79	72.83
2004	88.78	74.90	70.39	56.18	66.55	67.74	77.58	73.43
2005	86.84	74.79	70.69	55.70	66.72	68.68	75.29	73.49
2006	86.32	75.71	70.78	61.54	67.61	69.24	69.98	73.12
2007	84.69	75.92	68.90	60.87	71.10	70.08	69.30	71.52
2008	82.10	76.98	68.90	60.33	71.24	71.67	75.36	73.56
2009	83.27	77.87	68.34	59.65	72.46	71.65	73.49	73.07
2010	83.33	76.33	67.11	60.19	71.71	71.68	71.91	73.42
2011	83.88	76.69	66.53	63.50	70.99	70.87	73.78	73.42

2012	83.74	76.65	65.53	60.35	67.32	70.04	72.17	71.96
2013	83.61	75.61	66.65	60.01	66.25	70.66	74.21	71.38
2014	82.69	75.68	67.10	59.67	67.56	70.66	80.23	71.11
2015	82.37	75.62	68.19	58.65	67.14	69.74	79.72	71.81
2016	81.98	75.66	68.10	55.87	66.60	69.32	80.36	72.73
2017	82.09	73.59	69.14	57.33	66.14	69.50	79.94	72.20
2018	83.03	73.67	69.45	59.88	67.05	71.49	80.13	72.93
**Out-of-pocket health expenditure as % of current health expenditure**								
2000	10.20	20.37	27.33	47.66	27.15	31.12	10.84	n/d
2001	10.64	19.25	28.81	48.85	27.59	28.98	10.90	n/d
2002	10.00	20.56	27.51	47.58	25.60	26.45	11.02	n/d
2003	10.37	20.51	26.36	48.56	24.18	27.58	12.21	12.47
2004	10.71	22.41	25.83	40.63	32.92	29.44	20.91	12.16
2005	11.09	22.64	25.76	41.69	32.78	27.75	23.61	13.02
2006	11.65	23.17	25.01	35.60	31.85	27.11	26.59	12.27
2007	13.63	22.65	26.26	36.81	28.41	26.29	27.37	13.63
2008	16.13	20.72	26.35	37.31	28.17	24.43	21.02	12.64
2009	15.05	20.34	26.24	38.78	26.82	24.36	22.42	12.78
2010	15.25	21.92	27.42	37.19	27.59	23.71	22.80	12.64
2011	15.02	21.56	28.22	34.30	28.22	23.96	23.57	12.20
2012	15.29	21.52	29.37	37.82	31.80	24.26	23.23	12.46
2013	13.59	22.61	28.36	38.47	32.82	23.65	23.32	12.46
2014	14.08	22.62	28.34	39.11	31.49	23.13	18.01	12.98
2015	14.83	22.77	27.47	40.45	31.84	23.04	18.44	12.47
2016	15.02	22.69	27.67	43.32	32.32	22.82	18.19	11.98
2017	14.81	24.81	26.99	41.79	32.65	22.81	18.71	12.32
2018	14.19	24.56	26.89	39.18	31.64	20.42	18.91	11.93

Figure S1: Current health expenditure as % of GDP in Czechia, 2000-2018.

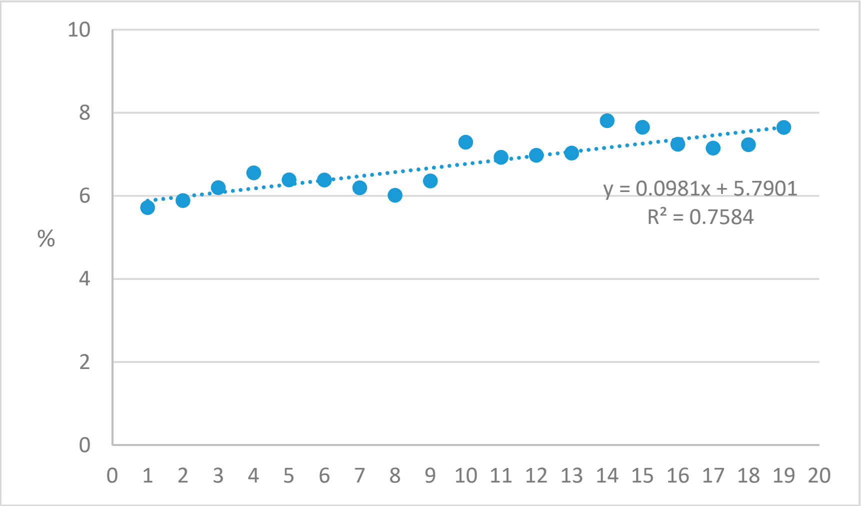

Figure S2: Current health expenditure as % of GDP in Estonia, 2000-2018.

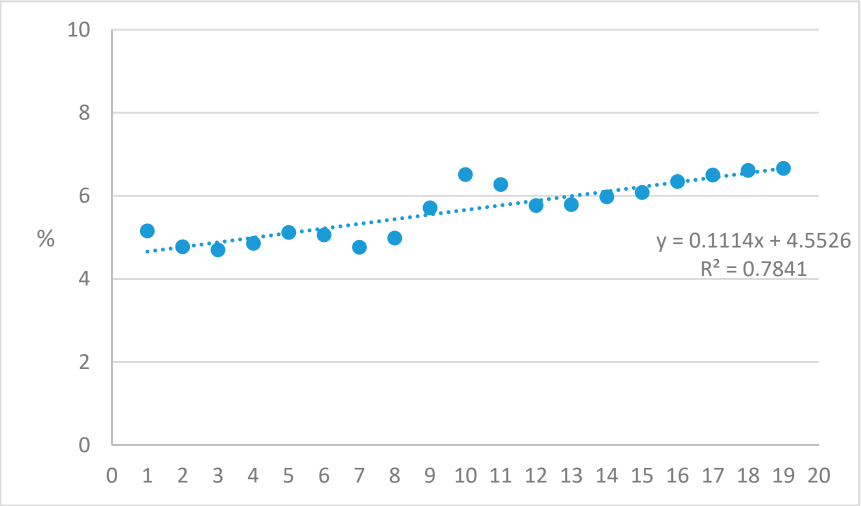

Figure S3: Current health expenditure as % of GDP in Hungary, 2000-2018.

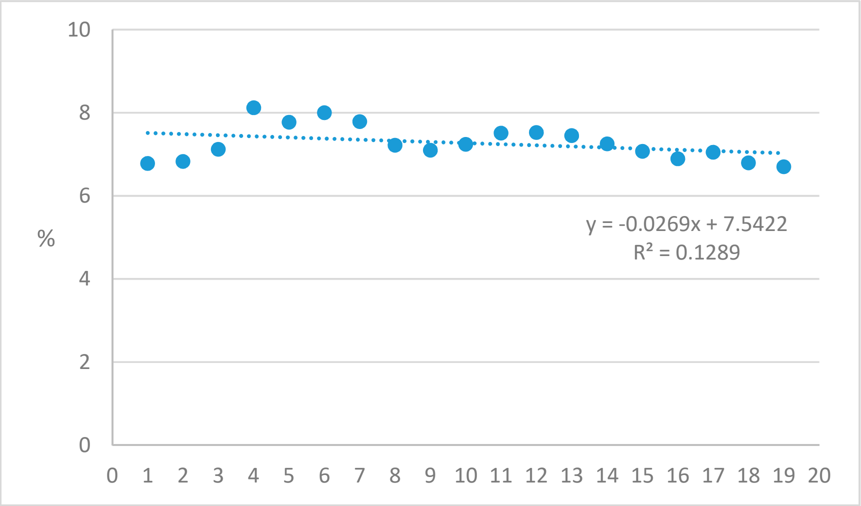

Figure S4: Current health expenditure as % of GDP in Latvia, 2000-2018.

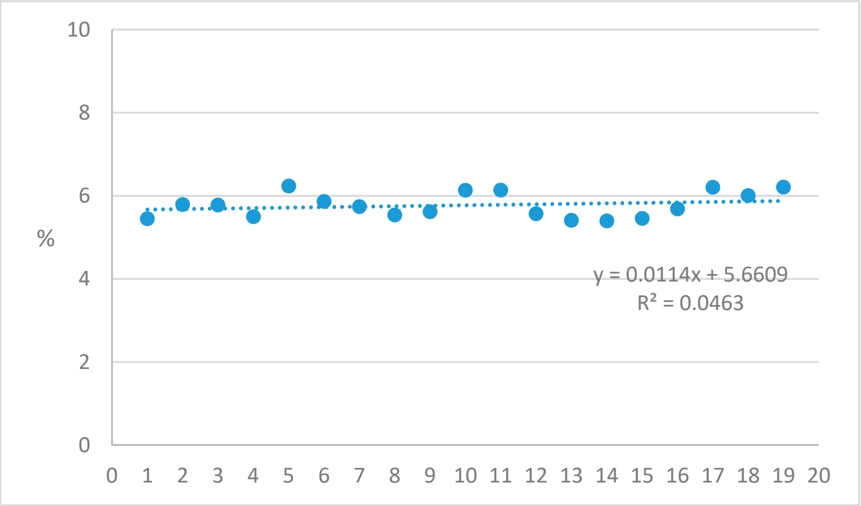

Figure S5: Current health expenditure as % of GDP in Lithuania, 2000-2018.

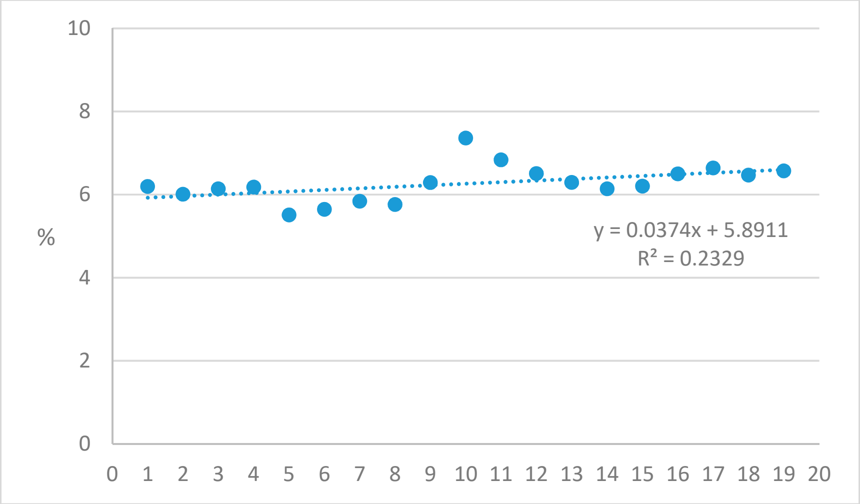

Figure S6: Current health expenditure as % of GDP in Poland, 2000-2018.

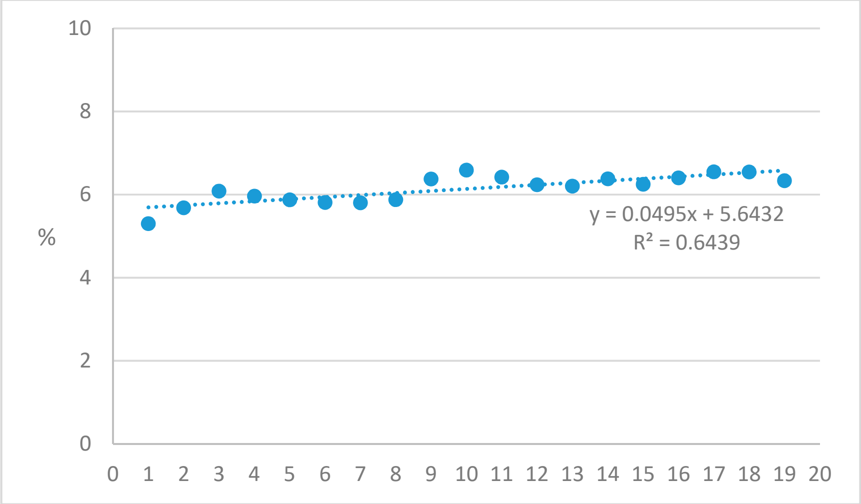

Figure S7: Current health expenditure as % of GDP in Slovakia, 2000-2018.

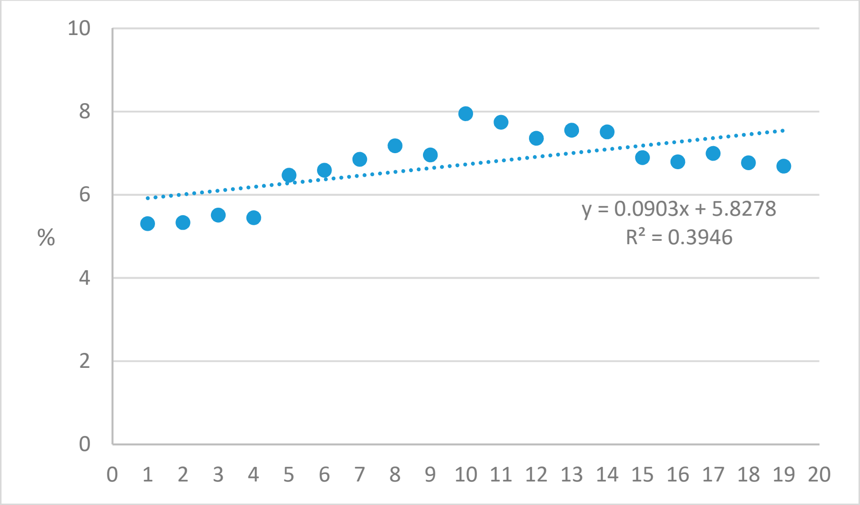

Figure S8: Current health expenditure as % of GDP in Slovenia, 2000-2018.

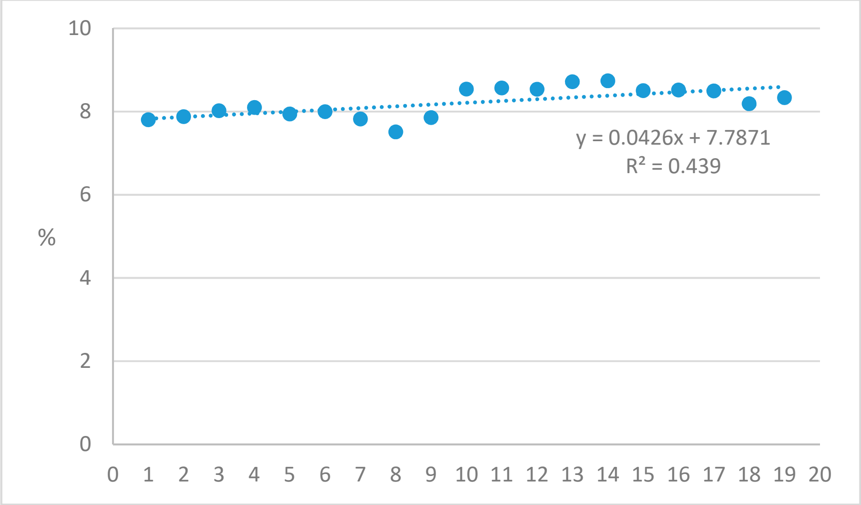

Figure S9: Domestic general government health expenditure as % of general government expenditure in Czechia, 2000-2018.

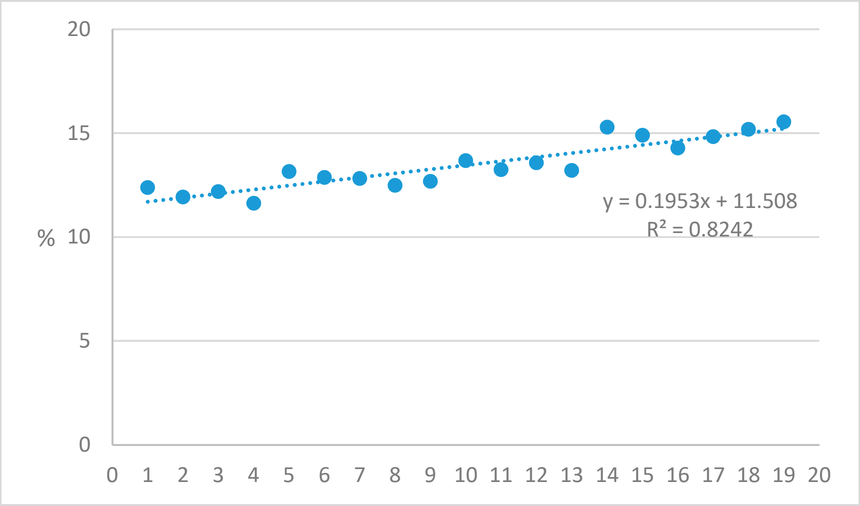

Figure S10: Domestic general government health expenditure as % of general government expenditure in Estonia, 2000-2018.

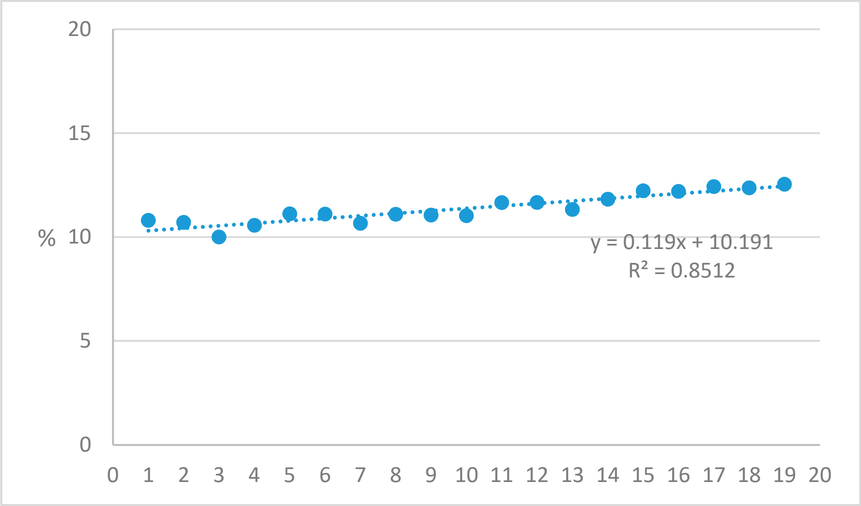

Figure S11: Domestic general government health expenditure as % of general government expenditure in Hungary, 2000-2018.

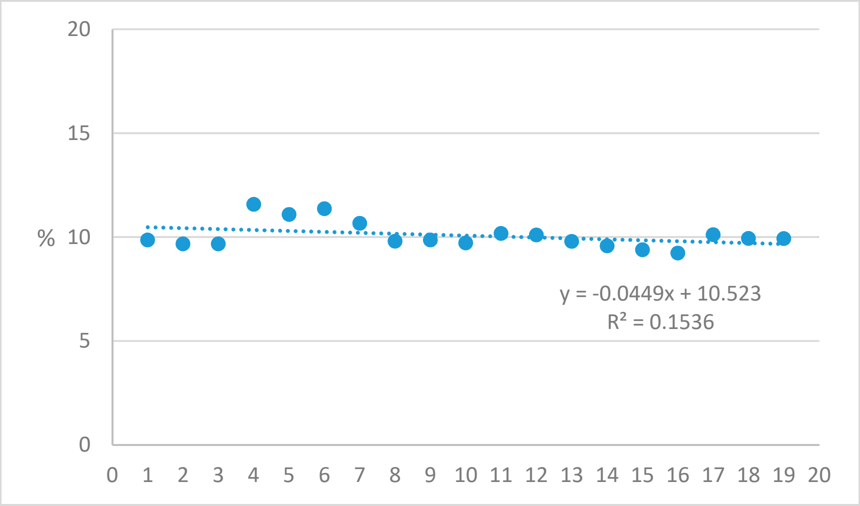

Figure S12: Domestic general government health expenditure as % of general government expenditure in Latvia, 2000-2018.

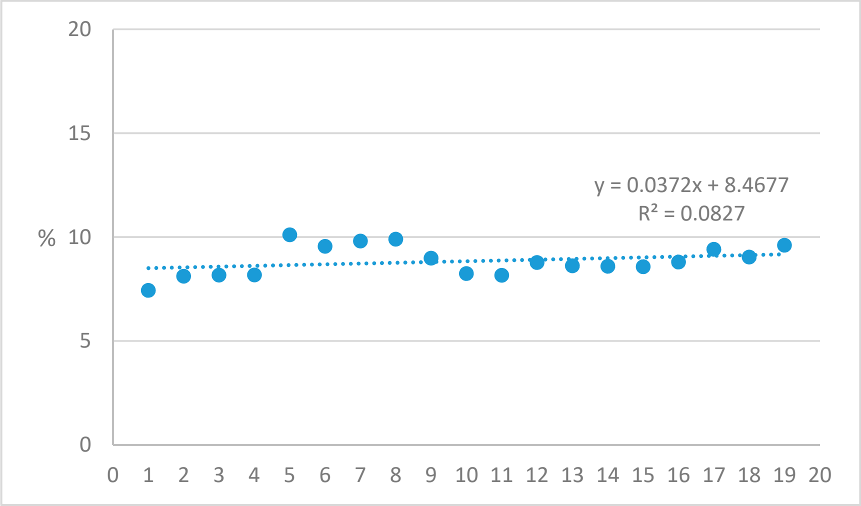

Figure S13: Domestic general government health expenditure as % of general government expenditure in Lithuania, 2000-2018.

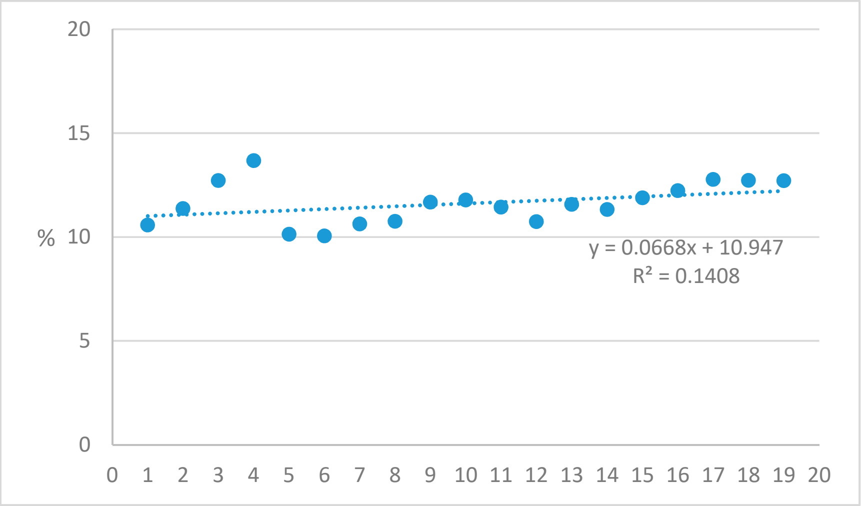

Figure S14: Domestic general government health expenditure as % of general government expenditure in Poland, 2000-2018.

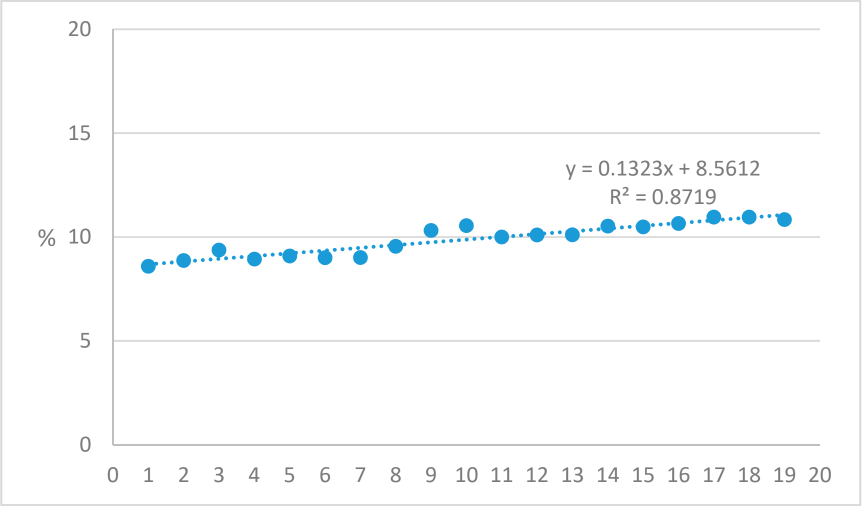

Figure S15: Domestic general government health expenditure as % of general government expenditure in Slovakia, 2000-2018.

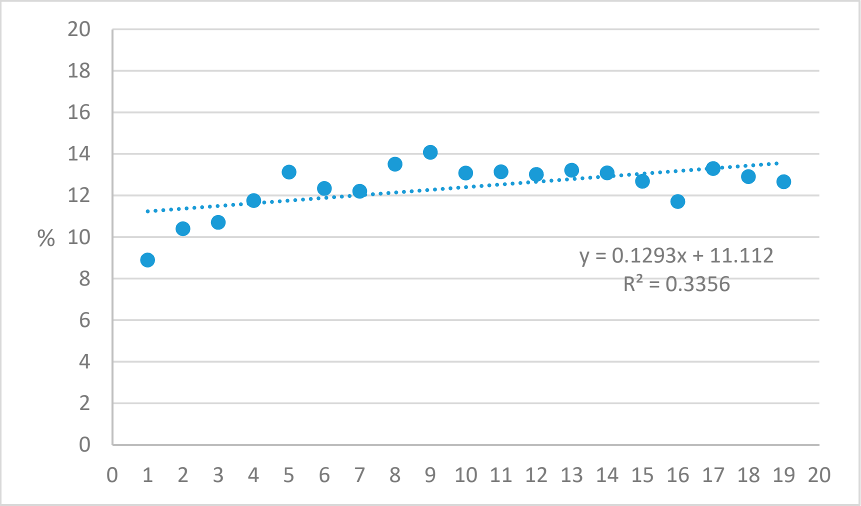

Figure S16: Domestic general government health expenditure as % of general government expenditure in Slovenia, 2000-2018.

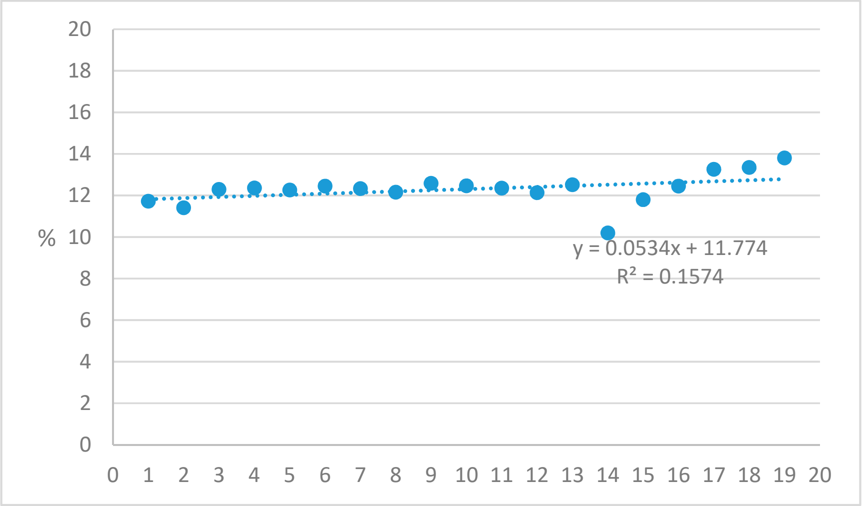

Figure S17: Government/compulsory scheme expenditure as % of current health expenditure in Czechia, 2000-2018.

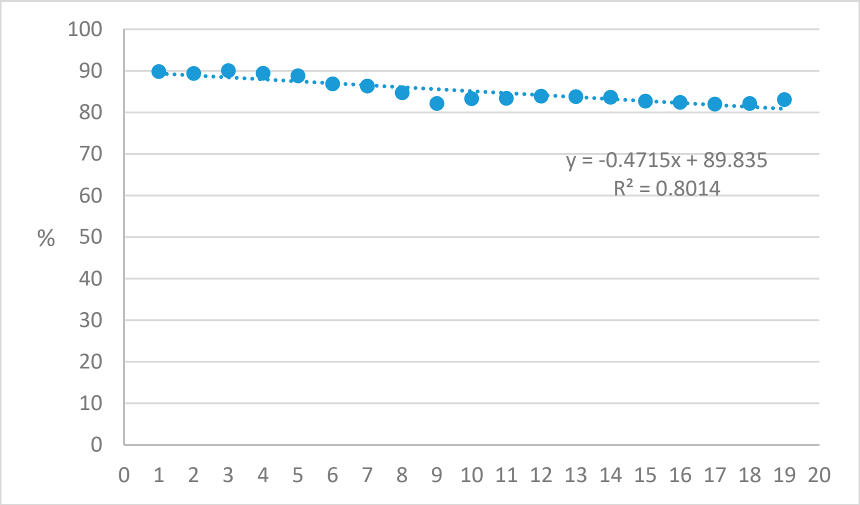

Figure S18: Government/compulsory scheme expenditure as % of current health expenditure in Estonia, 2000-2018.

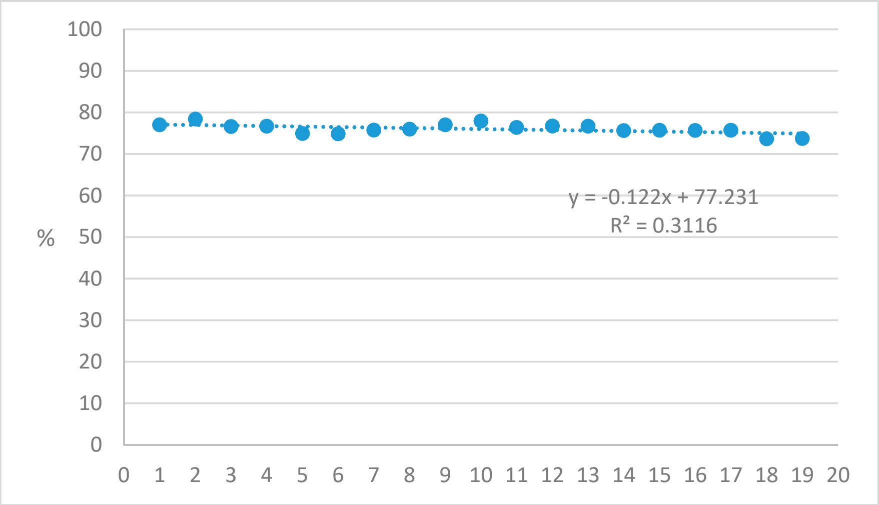

Figure S19: Government/compulsory scheme expenditure as % of current health expenditure in Hungary, 2000-2018.

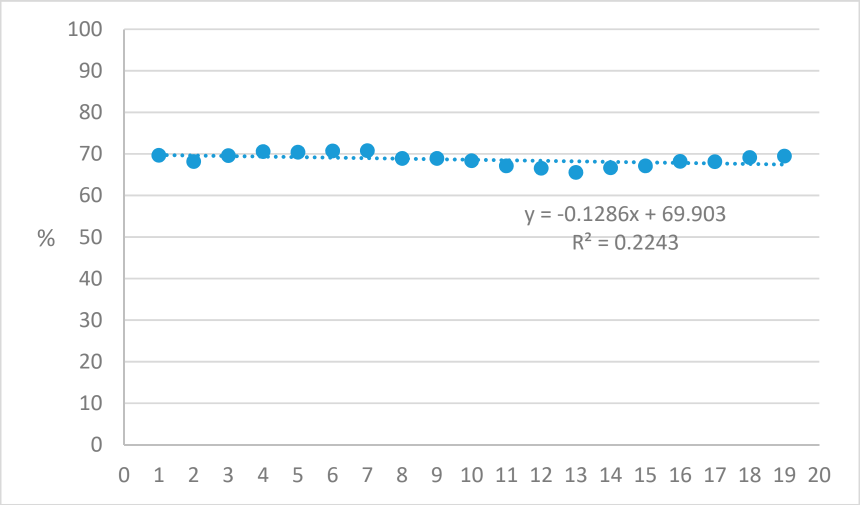

Figure S20: Government/compulsory scheme expenditure as % of current health expenditure in Latvia, 2000-2018.

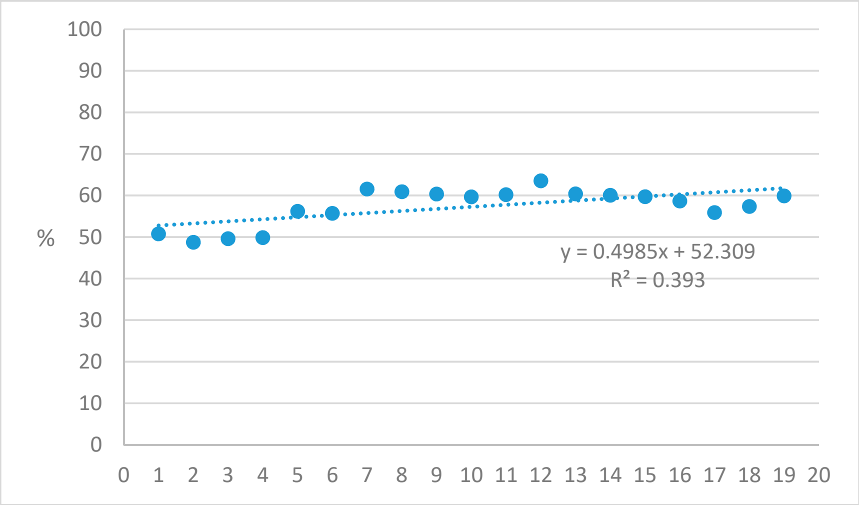

Figure S21: Government/compulsory scheme expenditure as % of current health expenditure in Lithuania, 2000-2018.

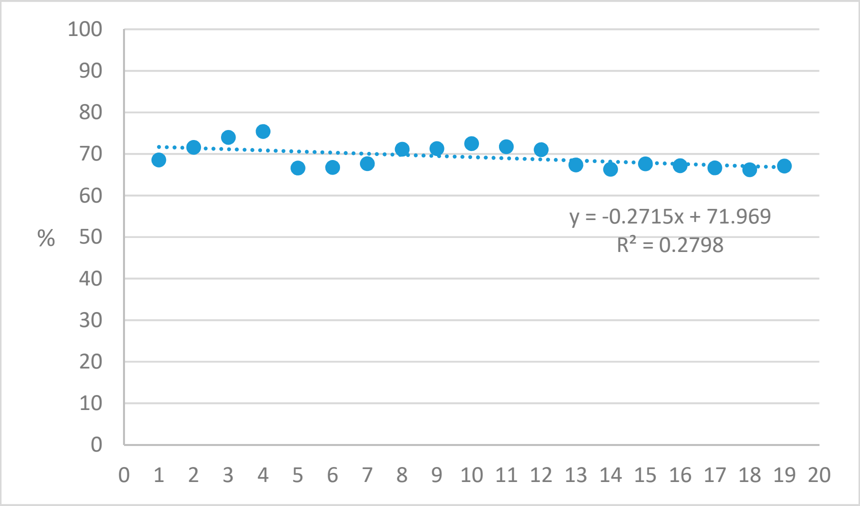

Figure S22: Government/compulsory scheme expenditure as % of current health expenditure in Poland, 2000-2018.

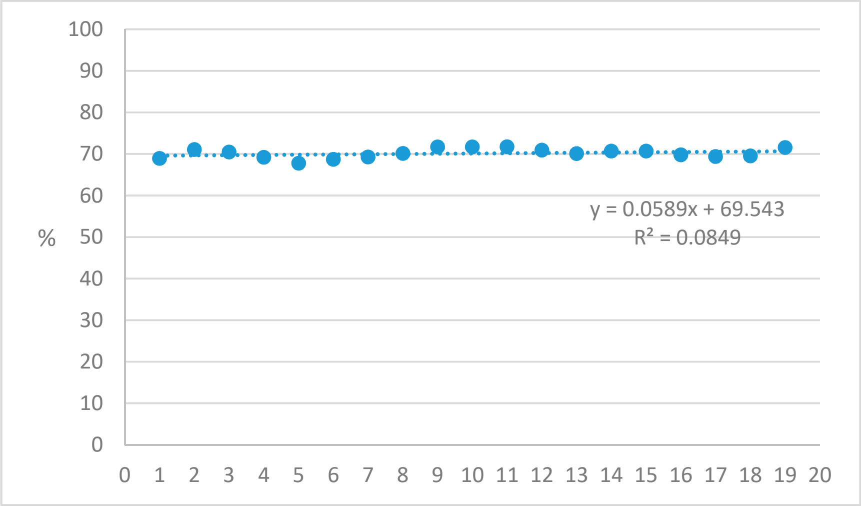

Figure S23: Government/compulsory scheme expenditure as % of current health expenditure in Slovakia, 2000-2018.

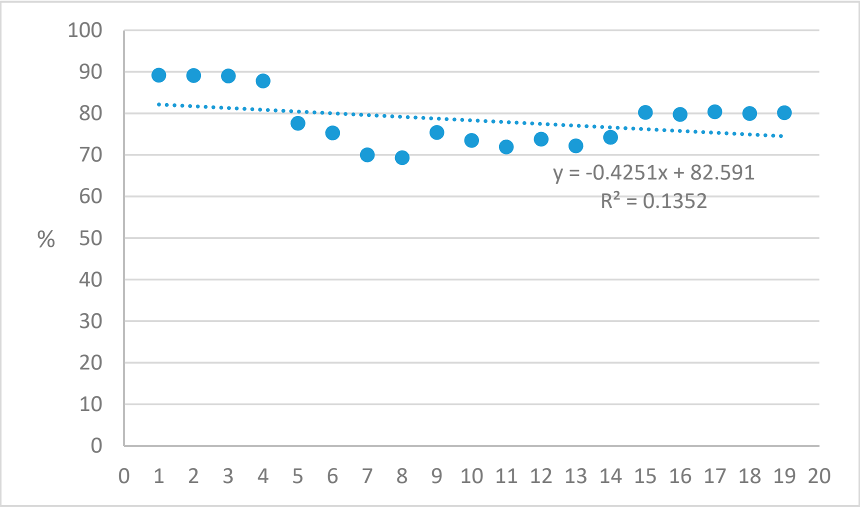

Figure S24: Government/compulsory scheme expenditure as % of current health expenditure in Slovenia, 2000-2018.

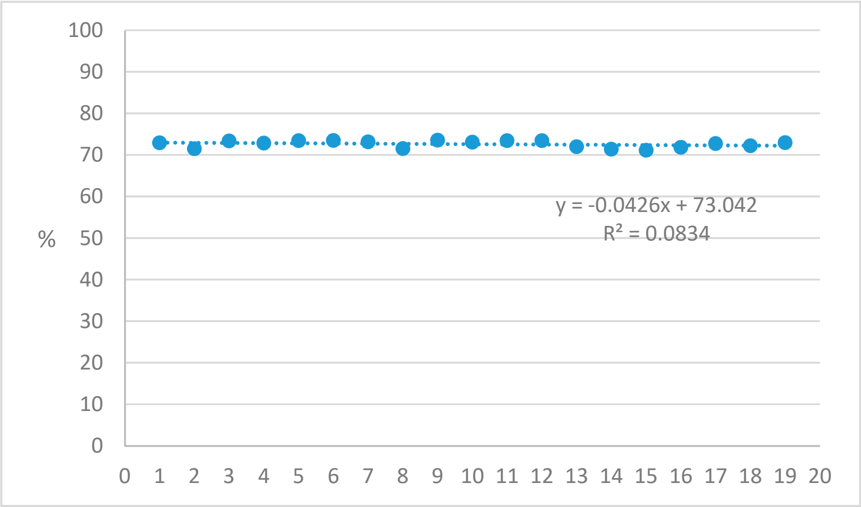

Figure S25: Out-of-pocket health expenditure as % of current health expenditure in Czechia, 2000-2018.

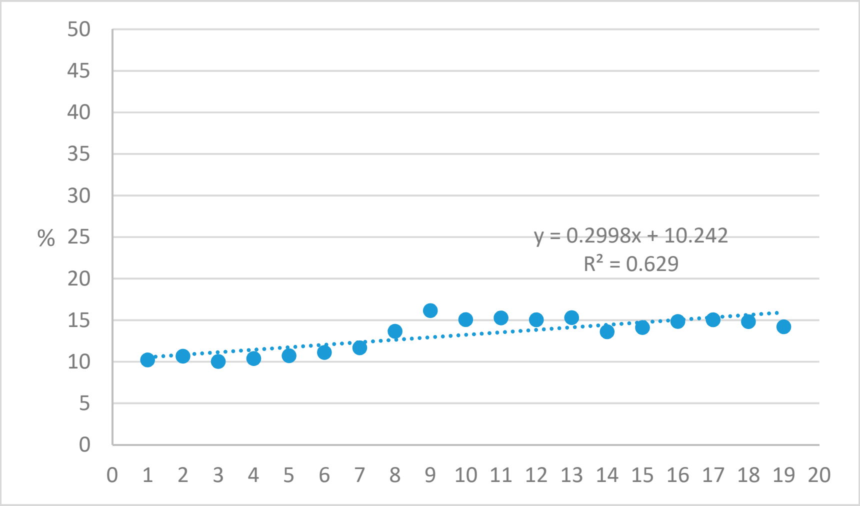

Figure S26: Out-of-pocket health expenditure as % of current health expenditure in Estonia, 2000-2018.

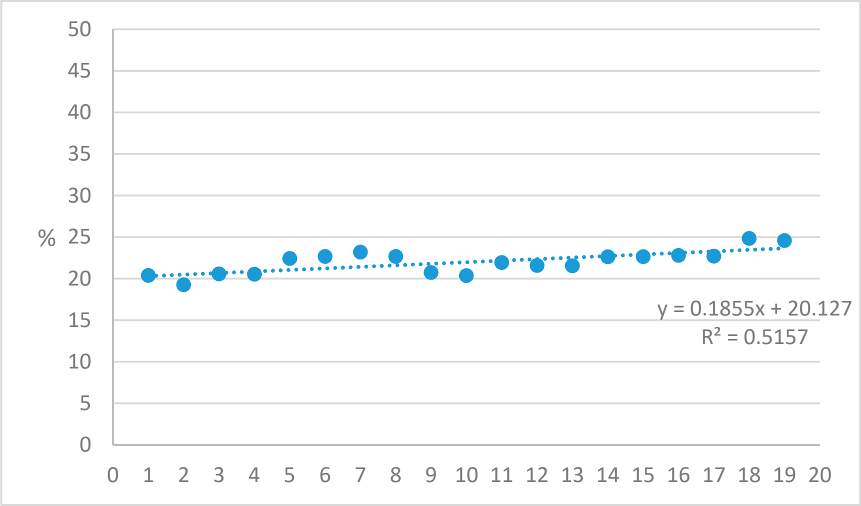

Figure S27: Out-of-pocket health expenditure as % of current health expenditure in Hungary, 2000-2018.

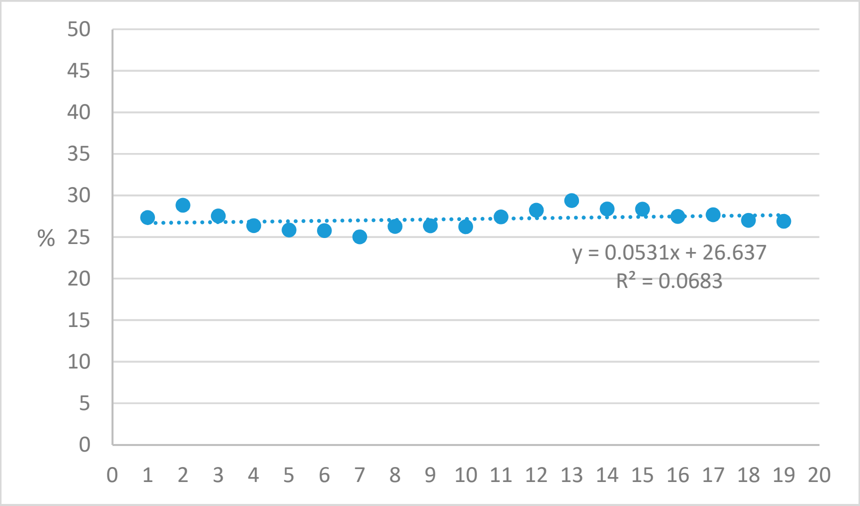

Figure S28: Out-of-pocket health expenditure as % of current health expenditure in Latvia, 2000-2018.

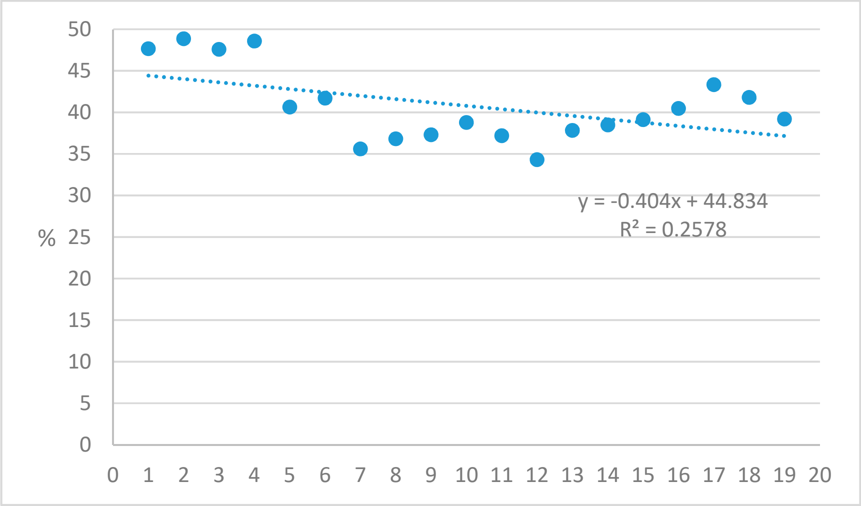

Figure S29: Out-of-pocket health expenditure as % of current health expenditure in Lithuania, 2000-2018.

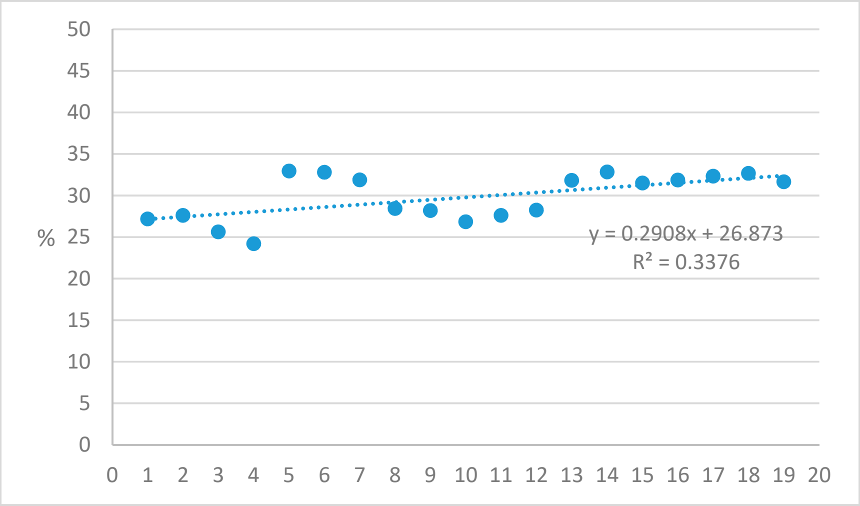

Figure S30: Out-of-pocket health expenditure as % of current health expenditure in Poland, 2000-2018.

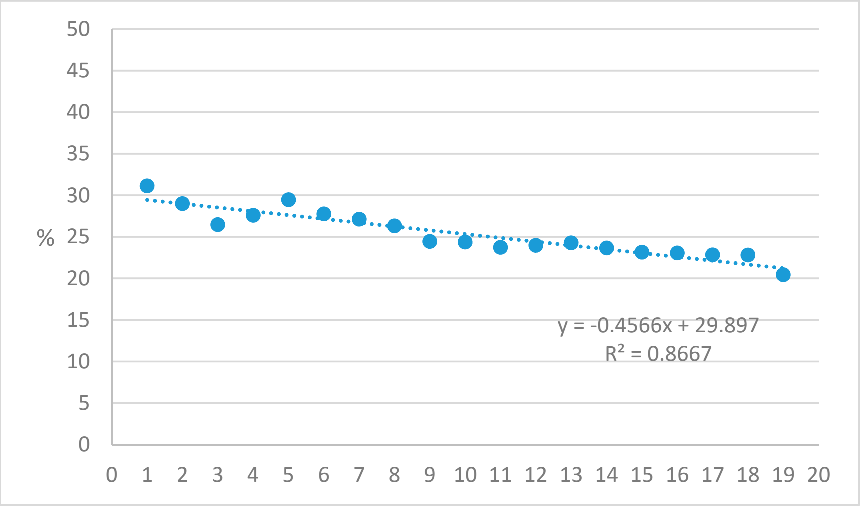

Figure S31: Out-of-pocket health expenditure as % of current health expenditure in Slovakia, 2000-2018.

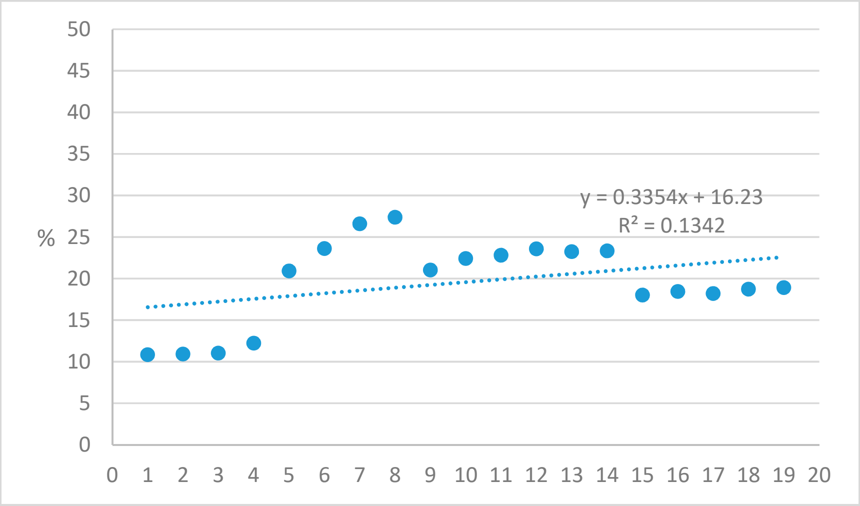

Figure S32: Out-of-pocket health expenditure as % of current health expenditure in Slovenia, 2000-2018.

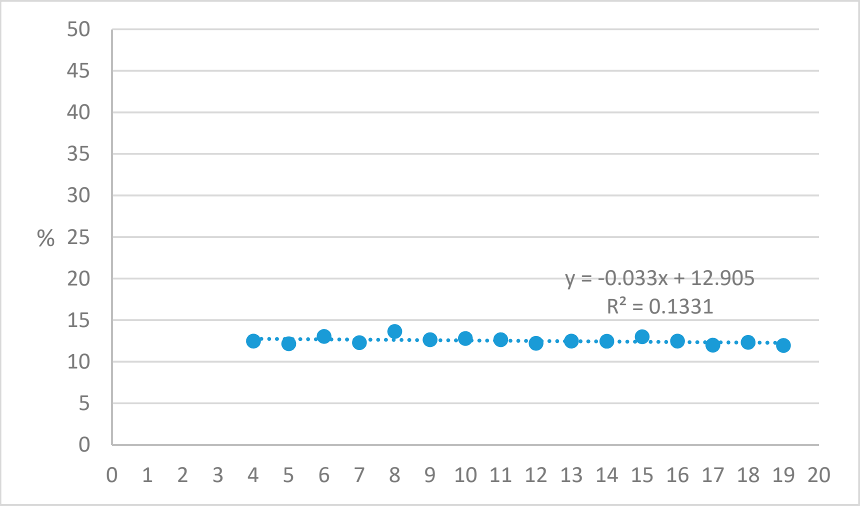

Supplement: Supplementary file 1 [file ijerph-18-01382-s001.pdf]
